# Explainable machine learning reveals multifactorial drivers of early intracranial hematoma progression in traumatic brain injury: development of a SHAP-guided SVM nomogram

**DOI:** 10.3389/fneur.2026.1718794

**Published:** 2026-02-05

**Authors:** Xujie Wang, Rongfei Xie, Minmin Li, Ziyi Zhao, Zhaohui Liu, Biyun Wang, Xuhui Liu

**Affiliations:** 1 Department of Emergency ICU, The Affiliated Hospital of Qinghai University, Xining, Qinghai, China; 2 Anhui North Coal and Electricity Group General Hospital, Suzhou, Anhui, China; 3 Department of Orthopedics, The First Medical Center of PLA General Hospital, Beijing, China; 4 Clinical Medical College of Tianjin Medical University, Tianjin, China; 5 Department of Biobank, Sun Yat-sen Memorial Hospital, Sun Yat-sen University, Guangzhou, China; 6 Department of Neurology, The Second Hospital of Lanzhou University, Lanzhou, Gansu, China

**Keywords:** hematoma progression, machine learning, nomogram, SHAP, support vector machine, traumatic brain injury

## Abstract

**Background:**

Early intracranial hematoma progression is a common and life-threatening complication of traumatic brain injury (TBI), associated with rapid neurological deterioration and poor outcomes. Accurate early identification of patients at risk remains challenging due to the multifactorial and nonlinear nature of underlying mechanisms. This study aimed to develop and validate an interpretable machine learning (ML) model for predicting early hematoma progression in TBI patients.

**Methods:**

We retrospectively analyzed clinical data from 356 patients with TBI admitted to Qinghai University Affiliated Hospital. Patients were randomly divided into training (70%) and internal validation (30%) cohorts. A total of 25 demographic, radiological, and laboratory variables were evaluated. Predictive features were selected using least absolute shrinkage and selection operator (LASSO) regression and further confirmed by multivariable logistic regression. Five ML algorithms were constructed and compared. The optimal model was interpreted using Shapley additive explanations (SHAP), followed by the development of a nomogram. Performance evaluation and risk-stratification analyses based on both model-derived probability estimates and nomoscore stratification were performed to assess the clinical utility of the model.

**Results:**

Early hematoma progression occurred in 49.7% (177/356) of patients. LASSO and logistic regression identified seven independent predictors: hematoma type, smoking history, age, D-dimer, monocyte-to-lymphocyte ratio (MLR), serum calcium, and multiple hematomas. Among the five algorithms, the support vector machine (SVM) achieved the best discrimination (training AUC = 0.937; validation AUC = 0.925), outperforming logistic regression, decision tree, XGBoost, and LightGBM. SHAP analysis confirmed the above variables as key contributors. The nomogram demonstrated strong predictive performance and interpretability. Rationality analyses showed that both model probability and nomoscore stratification exhibited stepwise increases in progression risk, validating the clinical robustness of the SVM-based model.

**Conclusion:**

We developed and validated an interpretable SVM model that accurately predicts early hematoma progression in TBI patients. By integrating demographic, radiological, and laboratory features, this model provides a reliable tool for early risk stratification, guiding individualized management and timely intervention. Its strong performance across subgroups underscores its clinical applicability.

## Introduction

Traumatic brain injury (TBI) is a devastating neurological disorder caused by external mechanical forces, leading to structural damage or functional impairment of the brain. Patients with TBI often present with impaired consciousness, memory loss, neurological deficits, and radiologically confirmed intracranial lesions (1). Globally, TBI is recognized as a major public health problem, affecting more than 50 million people annually and ranking as the leading cause of death and disability among young adults (2, 3). In the United States, approximately 1.7 million individuals sustain TBI each year, with more than 275,000 hospitalizations and over 50,000 deaths (4). In China, the annual incidence is estimated to be 770,000–890,000, making TBI one of the most prevalent causes of long-term disability and socioeconomic burden (5). Despite advances in emergency care, the case fatality rate of severe TBI remains as high as 30–40%, with more than 60% of survivors experiencing permanent neurological, cognitive, or psychosocial sequelae (6). Secondary brain injury, particularly early intracranial hematoma progression, is one of the most critical determinants of poor outcomes, increasing the risk of neurological deterioration fivefold and significantly prolonging hospitalization (7). Epidemiological studies indicate that the incidence of early hematoma progression ranges from 21 to 64% within the first 24 h after trauma (7, 8). Therefore, an accurate early prediction tool could help tailor the timing of follow-up CT scans, intensify clinical monitoring for high-risk patients, and prompt timely neurosurgical evaluation and decision-making.

The pathophysiology of hematoma progression is multifactorial, involving cerebral hypoxia, dysregulated vascular autoregulation, coagulopathy, inflammatory cascades, and blood–brain barrier disruption (9, 10). Previous studies have identified several risk factors, such as age, sex, initial hematoma volume, Glasgow Coma Scale (GCS) score, and coagulation abnormalities (7). In addition, comorbidities such as hypertension and diabetes, as well as laboratory parameters including white blood cell counts, platelet counts, calcium, and D-dimer, have been implicated in hematoma expansion (11, 12). However, the predictive ability of conventional regression-based models remains limited. These models often assume linear relationships, struggle with high-dimensional data, and fail to fully capture the complex nonlinear interactions underlying hematoma progression (13).

By contrast, machine learning (ML) algorithms provide a promising alternative, capable of handling nonlinear relationships and extracting hidden patterns from complex, high-dimensional datasets (14). In recent years, ML has been successfully applied in outcome prediction across multiple neurological and critical care conditions, including spontaneous intracerebral hemorrhage, ischemic stroke, sepsis, and traumatic brain injury (15–17). Support vector machines (SVM), gradient boosting algorithms, and ensemble methods have demonstrated superior predictive performance compared with traditional approaches (18). Nevertheless, studies focusing specifically on early hematoma/contusion progression after TBI remain relatively limited and heterogeneous, and the available prediction approaches are not yet sufficiently validated for routine bedside use (8, 19). However, early intracranial hematoma progression represents a uniquely actionable endpoint: a bedside prediction model applied at initial presentation could generate individualized risk estimates and thereby help tailor the timing of follow-up CT imaging, the intensity of clinical monitoring, and the need for timely neurosurgical evaluation. In particular, many existing approaches rely on linear assumptions or limited feature integration, and often provide insufficient interpretability or bedside-ready risk stratification.

Given the limited and heterogeneous evidence, and the difficulty of capturing nonlinear relationships within high-dimensional clinical, laboratory, and radiological predictors, we propose a clinically oriented, explainable modeling framework with comprehensive evaluation (discrimination, calibration, and decision-analytic utility) and nomogram/nomoscore-based risk stratification. In this context, we aimed to develop and internally validate ML-based predictive models for early intracranial hematoma progression in TBI patients and to evaluate their utility for early risk stratification to support clinical management. Compared with conventional regression, ML can better accommodate nonlinear contributions and complex joint effects among radiological, coagulation, inflammatory, and metabolic markers. We compared five commonly used ML algorithms under a unified feature set and evaluation framework (discrimination, calibration, and decision-analytic utility), and emphasized clinical translation by integrating SHAP-based explainability with a nomogram/nomoscore risk stratification scheme to provide transparent, bedside-oriented decision support. Specifically, our objectives were to compare model performance across algorithms, select the most optimal approach, and provide an interpretable, bedside-oriented tool that translates model outputs into clinically meaningful risk stratification and bridges statistical prediction with clinical decision-making.

## Methods

**Figure 1:**
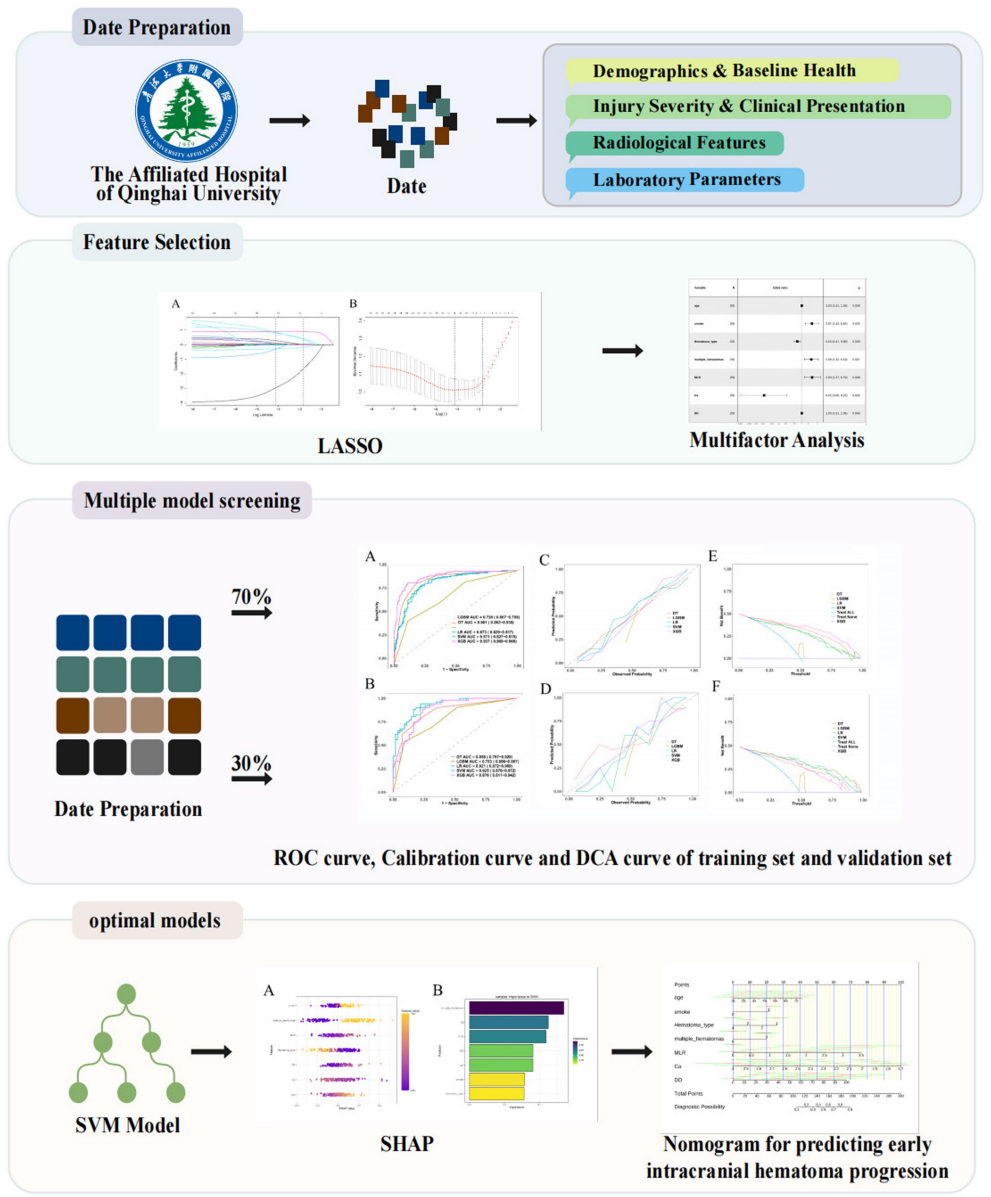
Flow diagram of the selection of eligible traumatic brain injury patients.

This retrospective observational study was conducted at Qinghai University Affiliated Hospital, China, in accordance with the RECORD (REporting of studies Conducted using Observational Routinely-collected health Data) guidelines. Clinical and imaging data of patients with traumatic brain injury (TBI) admitted between August 2022 and December 2023 were extracted from the hospital’s electronic medical record system. A total of 356 patients were enrolled after applying strict inclusion and exclusion criteria. The study protocol was reviewed and approved by the Ethics Committee of Qinghai University Affiliated Hospital (Approval No: P-SL-2025-265). Considering that this was a retrospective analysis of anonymized data with no identifiable patient information, the requirement for informed consent was waived. All study procedures complied with institutional and national standards for privacy and confidentiality. A consolidated study flow diagram summarizing screening, exclusions, and cohort allocation is provided in Figure 1.

The study population comprised patients aged 18 years or older who were admitted within 6 h after trauma, with cranial computed tomography (CT) or magnetic resonance imaging (MRI) confirming the presence of intracranial hematoma and a repeat cranial CT performed within 24 h. Inclusion criteria were: (1) age ≥18 years; (2) admission within 6 h after trauma; (3) initial CT or MRI confirming intracranial hematoma; (4) repeat cranial CT within 24 h of the index scan. Exclusion criteria were: (1) immediate neurosurgical intervention after the first CT; (2) known coagulopathies or current anticoagulation likely to affect coagulation; (3) active malignancy; (4) severe psychiatric or cognitive disorders precluding cooperation; (5) non-traumatic intracranial hemorrhage. For patients with multiple admissions, only the first admission was considered. Patients with more than 5% missing variables were excluded, whereas those with ≤5% missingness were imputed using the K-nearest neighbor (KNN) algorithm. The extent and pattern of missing data across all candidate variables are summarized in Supplementary Figure S1. Overall missingness was low and was confined to a small subset of laboratory variables, with the highest missingness observed for fibrinogen (15/356, 4.21%), followed by blood glucose (13/356, 3.65%), platelet count (12/356, 3.37%), and potassium (10/356, 2.81%) (Supplementary Figure S1). After screening, 356 patients were included and randomly allocated in a 70:30 ratio into a training cohort (*n* = 250) and a validation cohort (*n* = 106) to enable model development and internal validation.

The primary outcome of interest was early intracranial hematoma progression, defined radiologically as a hematoma volume increase of more than 33% or an absolute growth greater than 6 mL, the appearance of new hematoma lesions, enlargement of epidural or subdural hematomas leading to midline shift greater than 5 mm or basal cistern compression, or progression of posterior fossa hematomas with evidence of fourth ventricle obstruction. A total of 21 candidate predictors were collected, encompassing demographic factors (age, sex, smoking, hypertension, diabetes), injury-related variables (mechanism of injury, Glasgow Coma Scale [GCS], pupil reactivity), radiological features (hematoma type, multiplicity, heterogeneity), and laboratory parameters (blood glucose, white blood cell count, monocyte count, lymphocyte count, monocyte-to-lymphocyte ratio [MLR], platelet count, calcium, potassium, prothrombin time [PT], activated partial thromboplastin time [APTT], international normalized ratio [INR], fibrinogen, and D-dimer).

Univariate analyses were first conducted using χ^2^ or Fisher’s exact tests for categorical variables and Mann–Whitney U tests for continuous variables. Variables with *p* < 0.05 were further screened using the least absolute shrinkage and selection operator (LASSO) logistic regression with 10-fold cross-validation to minimize overfitting. The optimal penalty parameter (*λ* = 0.0595) was selected based on the one-standard error (1-SE) rule, yielding seven predictors with non-zero coefficients. These predictors were then entered into a multivariate logistic regression model with backward stepwise selection, and all retained variables were confirmed as independent predictors of hematoma progression. Variance inflation factors (VIF) were assessed, and no significant multicollinearity was detected.

The seven independent predictors (age, smoking history, hematoma type, multiple hematomas, MLR, serum calcium, and D-dimer) were used to construct five machine learning models, namely logistic regression (LR), support vector machine (SVM), decision tree (DT), eXtreme Gradient Boosting (XGB), and Light Gradient Boosting Machine (LightGBM). The performance of each model was comprehensively evaluated in terms of discrimination, calibration, and clinical utility. Discrimination was quantified using the area under the receiver operating characteristic curve (AUC), precision–recall analysis, and confusion-matrix–based indices including sensitivity, specificity, precision, recall, F1 score, positive predictive value (PPV), and negative predictive value (NPV). Calibration was evaluated by calibration plots, the Hosmer–Lemeshow goodness-of-fit test, and Brier scores. Clinical applicability was assessed by decision curve analysis (DCA), and area under the precision–recall curve (AUPRC) was additionally employed in subgroup analyses to account for potential class imbalance.

Among the five models, the SVM classifier exhibited the most robust and generalizable performance, with consistently high accuracy, sensitivity, and specificity across both cohorts. To enhance interpretability, Shapley additive explanations (SHAP) were applied to the SVM model, enabling quantification of the contribution of each predictor to the model output. The SHAP summary plot revealed that hematoma type, smoking, and age contributed most strongly to the prediction, followed by D-dimer, MLR, calcium, and multiplicity of hematomas. Higher age, smoking history, elevated D-dimer, increased MLR, and multiple hematomas were associated with elevated progression risk, whereas higher serum calcium exerted a protective effect, highlighting the multifactorial pathophysiology underlying hematoma expansion.

On the basis of the SVM classifier, we constructed a nomogram to facilitate individualized risk assessment. Each predictor was assigned a weighted score, and the total score corresponded to the estimated probability of progression, thereby generating a composite nomoscore. The nomogram enabled clinicians to integrate demographic, biochemical, and radiological predictors into a single quantitative tool for bedside decision-making. The diagnostic accuracy of the nomogram was high, with AUCs exceeding 0.90, sensitivity and specificity greater than 0.80, and favorable PPV and NPV in both training and validation cohorts. Calibration plots and DCA confirmed good agreement between predicted and observed outcomes and demonstrated superior net benefit compared with individual predictors.

To further evaluate the risk-stratification performance of the nomoscore, patients were stratified into quartiles based on total scores. A clear dose–response relationship was observed, with stepwise increases in progression risk from Q1 to Q4. Patients in the highest quartile exhibited more than a threefold increased risk of hematoma progression compared with those in the lowest quartile. This monotonic trend was validated across clinical subgroups, and graphical visualization using forest plots and boxplots further confirmed significantly higher nomoscores in patients who developed progression. These results support the nomoscore as a rational, interpretable, and clinically applicable scoring system derived from the SVM-based nomogram, effectively stratifying patients into distinct risk categories and bridging statistical prediction with bedside decision support.

Categorical variables were expressed as counts and percentages, while continuous variables were summarized as medians with interquartile ranges. Statistical comparisons between groups were conducted using χ^2^ or Fisher’s exact test for categorical variables and Mann–Whitney U test for continuous variables. Logistic regression models reported odds ratios (ORs) and 95% confidence intervals (CIs). All statistical analyses were conducted using R software (version 4.2.1) and Python (version 3.6.5), and two-tailed *p* < 0.05 was considered statistically significant.

## Results

A total of 356 patients with traumatic brain injury (TBI) were enrolled in this study, including 277 males (77.81%) and 79 females (22.19%), with a median age of 52.00 years (interquartile range [IQR]: 41.00–62.25). Among them, 250 patients were randomly assigned to the training cohort and 106 to the validation cohort. The overall incidence of early intracranial hematoma progression was 49.7% (*n* = 177). As shown in Table 1, baseline demographic and clinical characteristics were generally well balanced between the training and validation cohorts, with no significant differences in age, sex, comorbidities, injury mechanism, neurological status, imaging features, or key laboratory indices (all *p* > 0.05). In contrast, substantial differences were observed between patients with and without hematoma progression, as presented in Supplementary Table S1. Patients in the progression group were older (median age: 55.00 vs. 49.00 years, *p* < 0.001), more likely to be male (81.92% vs. 73.74%) and to have a history of smoking (49.72% vs. 22.35%, *p* < 0.001). Severe TBI (GCS ≤ 8) was more common among patients with progression (*p* = 0.005), as were radiological features such as multiple hematomas (74.58% vs. 25.70%) and hematoma heterogeneity (32.77% vs. 15.08%), both *p* < 0.001. Hematoma types also differed significantly between groups, with intracerebral hematoma more frequent in the progression group (62.15% vs. 33.52%, *p* < 0.001). Laboratory parameters revealed that patients with hematoma progression had significantly elevated white blood cell counts, monocyte counts, D-dimer levels, and monocyte-to-lymphocyte ratio (MLR), along with lower platelet counts and serum calcium concentrations (all *p* < 0.01). Admission glucose levels were also higher in this group (7.80 vs. 6.50 mmol/L, *p* < 0.001). Collectively, these findings suggest that both clinical and biochemical variables are associated with early hematoma progression in TBI patients and may serve as valuable candidates for predictive modeling.

**Table 1:** Baseline characteristics of the training and validation cohorts in patients with traumatic brain injury.

Variables	All (*N* = 356)	Test (*N* = 106)	Train (*N* = 250)	*P* value (overall)
Grouped	0.50 (0.50)	0.49 (0.50)	0.50 (0.50)	0.871
Gender				0.785
Male	277 (77.81%)	81 (76.42%)	196 (78.40%)	
Female	79 (22.19%)	25 (23.58%)	54 (21.60%)	
Age	52.00 [41.00;62.25]	49.50 [41.00;57.00]	54.00 [41.00;63.00]	0.143
Hypertension				0.608
No	270 (75.84%)	78 (73.58%)	192 (76.80%)	
Yes	86 (24.16%)	28 (26.42%)	58 (23.20%)	
Diabetes				0.021
No	304 (85.39%)	83 (78.30%)	221 (88.40%)	
Yes	52 (14.61%)	23 (21.70%)	29 (11.60%)	
Smoke				0.564
No	228 (64.04%)	65 (61.32%)	163 (65.20%)	
Yes	128 (35.96%)	41 (38.68%)	87 (34.80%)	
Systolic_pressure	123.00 [120.00;138.00]	129.00 [120.00;139.50]	121.50 [120.00;138.00]	0.358
Diastolic_pressure	80.00 [72.00;89.00]	80.00 [73.25;89.00]	80.00 [71.00;88.00]	0.567
Mechanism_of_injury				0.374
Traffic accident	105 (29.49%)	37 (34.91%)	68 (27.20%)	
Fall from height	76 (21.35%)	22 (20.75%)	54 (21.60%)	
Ground-level fall	149 (41.85%)	38 (35.85%)	111 (44.40%)	
Other	26 (7.30%)	9 (8.49%)	17 (6.80%)	
GCS				0.135
Mild	214 (60.11%)	71 (66.98%)	143 (57.20%)	
Moderate	68 (19.10%)	14 (13.21%)	54 (21.60%)	
Severe	74 (20.79%)	21 (19.81%)	53 (21.20%)	
Glu	7.30 [5.77;9.40]	7.30 [5.73;9.47]	7.25 [5.80;9.38]	0.726
Pupil_reaction				0.739
No	297 (83.43%)	90 (84.91%)	207 (82.80%)	
Yes	59 (16.57%)	16 (15.09%)	43 (17.20%)	
Hematoma_type				0.620
tICH	170 (47.75%)	51 (48.11%)	119 (47.60%)	
tSDH	64 (17.98%)	15 (14.15%)	49 (19.60%)	
tEDH	54 (15.17%)	18 (16.98%)	36 (14.40%)	
tSAH	68 (19.10%)	22 (20.75%)	46 (18.40%)	
Multiple_hematomas				0.297
No	178 (50.00%)	58 (54.72%)	120 (48.00%)	
Yes	178 (50.00%)	48 (45.28%)	130 (52.00%)	
Hematoma_heterogeneity				0.746
No	271 (76.12%)	79 (74.53%)	192 (76.80%)	
Yes	85 (23.88%)	27 (25.47%)	58 (23.20%)	
White_blood_cell_count	13.31 [9.87;16.67]	13.12 [10.02;17.88]	13.38 [9.65;16.56]	0.769
Monocyte_count	0.69 [0.51;0.94]	0.72 [0.55;1.06]	0.67 [0.49;0.92]	0.139
Lymphocyte_count	0.95 [0.69;1.29]	0.93 [0.65;1.26]	0.96 [0.69;1.32]	0.618
MLR	0.74 [0.48;1.10]	0.76 [0.55;1.27]	0.70 [0.47;1.05]	0.047
Plt	176.50 [145.00;210.25]	178.00 [141.00;211.75]	175.00 [145.00;208.00]	0.890
Ca	2.17 [2.07;2.28]	2.17 [2.07;2.28]	2.17 [2.07;2.28]	0.869
Ka	3.67 [3.38;3.92]	3.59 [3.35;3.87]	3.69 [3.40;3.98]	0.157
PT	11.40 [10.70;12.30]	11.35 [10.80;12.07]	11.40 [10.60;12.47]	0.636
APTT	25.40 [23.10;28.30]	25.45 [23.13;28.17]	25.20 [23.02;28.50]	0.898
INR	0.96 [0.89;1.02]	0.96 [0.90;1.01]	0.96 [0.89;1.03]	0.802
FIB	2.32 [1.94;3.01]	2.28 [1.94;2.98]	2.34 [1.94;3.01]	0.559
DD	9.95 [3.27;22.57]	10.20 [3.40;21.00]	9.75 [3.20;23.70]	0.785

GCS, Glasgow Coma Scale; Glu, blood glucose; SBP, systolic blood pressure; DBP, diastolic blood pressure; tICH, traumatic intracerebral hemorrhage; tSDH, traumatic subdural hematoma; tEDH, traumatic epidural hematoma; tSAH, traumatic subarachnoid hemorrhage; MLR, monocyte-to-lymphocyte ratio; Plt, platelet count; Ca, serum calcium; Ka, serum potassium; PT, prothrombin time; APTT, activated partial thromboplastin time; INR, international normalized ratio; FIB, fibrinogen; DD, D-dimer.

### Variable selection by LASSO regression

**Figure 2:**
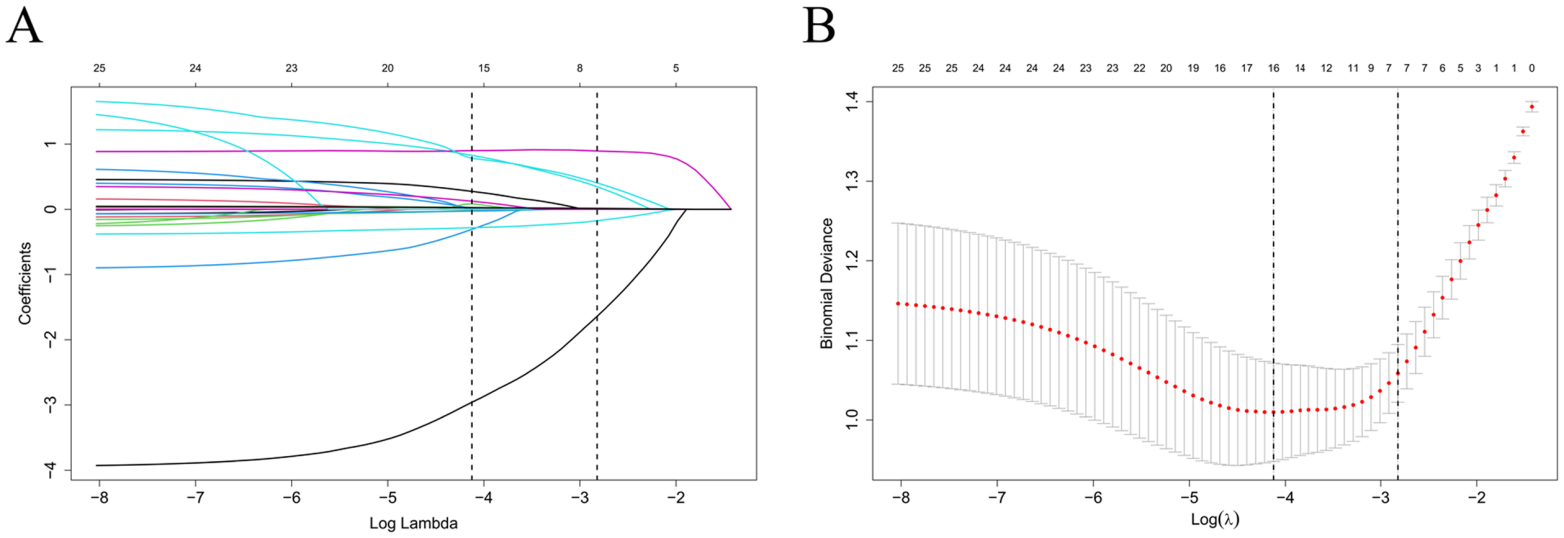
LASSO regression for feature selection. **(A)** Coefficient profiles of 21 candidate variables across log(*λ*). **(B)** Ten-fold cross-validation identifying optimal λ. The 1-SE criterion (λ = 0.0595) was used for model selection.

To identify the most predictive variables and avoid overfitting, we employed the least absolute shrinkage and selection operator (LASSO) logistic regression model with 10-fold cross-validation. The penalty parameter *λ* was tuned using both the minimum criteria and the 1-standard error (1-SE) rule. As shown in Figure 2A, the LASSO coefficient profiles were plotted against log(*λ*); with increasing penalization, most coefficients progressively shrank toward zero, illustrating the variable selection process. Figure 2B presents the cross-validated binomial deviance curve from the primary analysis cohort, with vertical dashed lines indicating the λ values corresponding to the minimum deviance (Lambda.min) and the 1-SE rule (Lambda.1se). For improved generalizability, we adopted the 1-SE criterion and selected Lambda.1se = 0.0595 as the optimal penalization point. A consistent pattern of deviance minimization across folds was observed in the extended analysis shown in Supplementary Figure S2, further validating the stability of our model selection process. At this optimal penalization level, a total of seven predictors with non-zero coefficients were retained in the final model (see Supplementary Table S2): age (*β* = 0.0067), smoking history (*β* = 0.3461), hematoma type (*β* = −0.1746), multiple hematomas (*β* = 0.8946), monocyte-to-lymphocyte ratio (MLR, *β* = 0.4044), serum calcium (*β* = −1.6408), and D-dimer (DD, *β* = 0.0151), along with the intercept term (*β* = 2.4040). These predictors collectively span demographic, radiological, and biochemical dimensions, highlighting the complex pathophysiology underlying early hematoma progression in patients with traumatic brain injury.

### Multivariate logistic regression identifies independent predictors of early hematoma progression

**Figure 3:**
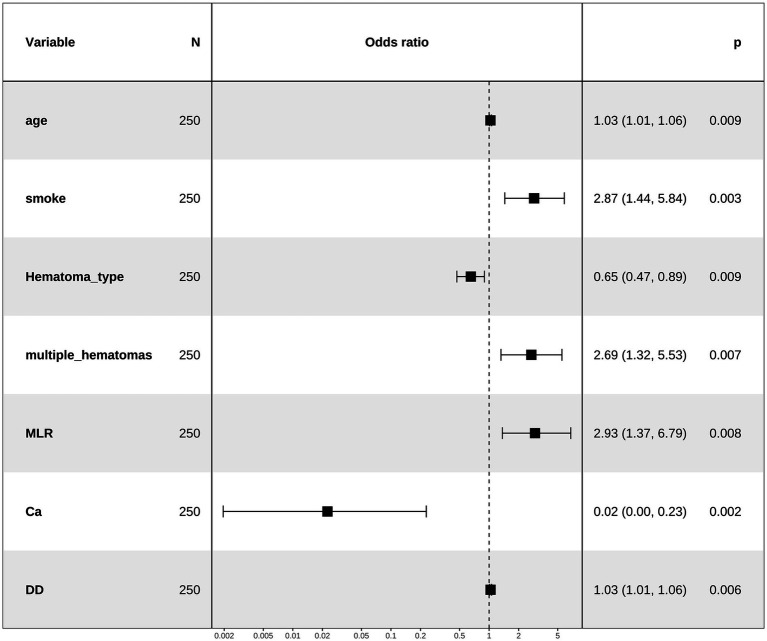
Forest plot of multivariate logistic regression analysis for early hematoma progression. The forest plot shows the odds ratios (ORs) and 95% confidence intervals (CIs) for predictors included in the final multivariate model. Variables significantly associated with early hematoma progression included age, smoking history, hematoma type, multiple hematomas, monocyte-to-lymphocyte ratio (MLR), serum calcium, and D-dimer.

To further identify independent predictors of early intracranial hematoma progression, all variables selected by the LASSO regression were entered into a multivariate logistic regression model using backward stepwise selection. As shown in Figure 3, seven variables remained significantly associated with the outcome. Advancing age was independently associated with increased risk (OR = 1.030, 95% CI: 1.007–1.054, *p* = 0.011). Patients with a history of smoking had over twofold greater odds of hematoma progression (OR = 2.294, 95% CI: 1.266–4.158, *p* = 0.006). The presence of multiple hematomas was a strong risk factor (OR = 4.385, 95% CI: 2.451–7.841, *p* < 0.001), as was a higher monocyte-to-lymphocyte ratio (MLR) (OR = 2.402, 95% CI: 1.163–4.959, *p* = 0.018). Elevated D-dimer levels were also significantly associated with progression (OR = 1.107 per unit increase, 95% CI: 1.015–1.209, *p* = 0.022), suggesting a pro-thrombotic and hyperfibrinolytic state. Conversely, higher serum calcium was independently associated with lower odds of hematoma progression (OR = 0.087, 95% CI: 0.018–0.419, *p* = 0.002), and intraparenchymal hematoma types were inversely associated with progression (OR = 0.479, 95% CI: 0.260–0.883, p = 0.018). These results support a multifactorial etiology involving demographic, inflammatory, coagulopathic, and radiological mechanisms. Multicollinearity was excluded using variance inflation factors (VIF), as summarized in Supplementary Table S3.

### Machine-learning model development and evaluation

**Figure 4:**
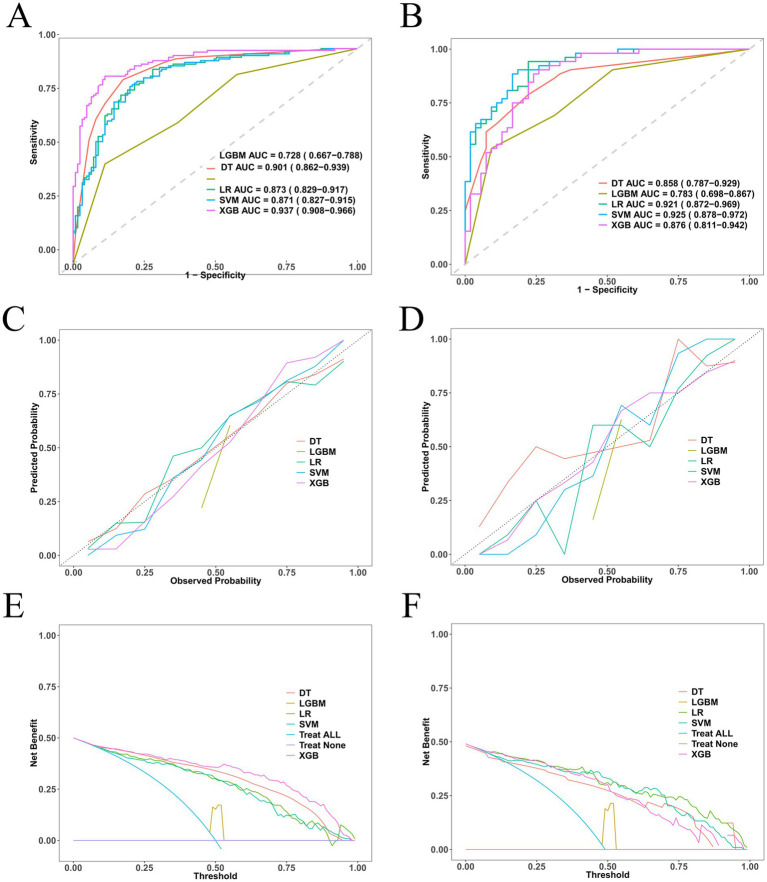
Performance of five machine-learning models for predicting early intracranial hematoma progression. **(A)** ROC curves in the training cohort. **(B)** ROC curves in the validation cohort. **(C)** Calibration plot in the training cohort. **(D)** Calibration plot in the validation cohort. **(E)** Decision-curve analysis (DCA) in the training cohort. **(F)** DCA in the validation cohort.

Using the seven independent predictors, we developed five machine learning models (LR, SVM, DT, XGB, and LGBM) and comprehensively assessed their performance in terms of discrimination, calibration, and clinical utility. In the training cohort, XGB achieved the highest discrimination (AUC = 0.937, 95% CI: 0.908–0.966), while DT, LR, and SVM also performed well, and LGBM showed comparatively lower accuracy. In the validation cohort, SVM (AUC = 0.925, 95% CI: 0.878–0.972) and LR (AUC = 0.921, 95% CI: 0.872–0.969) demonstrated the most robust generalization, whereas XGB, DT, and LGBM yielded moderate performance (Figures 4A,B, Table 2, and Supplementary Tables S4, S5). Beyond AUC, calibration analyses confirmed good agreement between predicted and observed probabilities (Figures 4C,D), and decision-curve analyses indicated that LR and SVM consistently provided the greatest net clinical benefit across a wide range of threshold probabilities (Figures 4E,F). Additional metrics, including precision–recall (PR) parameters (Supplementary Tables S6, S7 and Supplementary Figure S3), Brier scores (Supplementary Tables S8, S9), and confusion-matrix–based indicators such as sensitivity, specificity, predictive values, precision, recall, and F1 scores (Supplementary Figure S4), further validated the superior robustness of LR and SVM over the other algorithms. Direct comparisons of AUC distributions across models (Supplementary Table S10 and Supplementary Figure S5) reinforced these findings, showing that although XGB achieved excellent discrimination in the training cohort, LR and SVM offered the most consistent, reliable, and clinically applicable predictive performance in the validation cohort. Together, these results highlight LR and SVM as the most balanced and generalizable models, supported by convergent evidence across multiple evaluation metrics.

**Table 2:** Discrimination of five machine-learning models in the training and validation cohorts.

Model	dfclass	Sensitivity	Specificity	Pos Pred value	Neg Pred value	Precision	Recall	F1
LR	dev	0.904	0.72	0.764	0.882	0.764	0.904	0.828
DT	dev	0.829	0.851	0.856	0.824	0.856	0.829	0.843
XGB	dev	0.872	0.888	0.886	0.874	0.886	0.872	0.879
SVM	dev	0.848	0.776	0.791	0.836	0.791	0.848	0.819
LGBM	dev	0.464	0.888	0.806	0.624	0.806	0.464	0.589
LR	vad	0.942	0.685	0.742	0.925	0.742	0.942	0.831
DT	vad	0.774	0.792	0.788	0.778	0.788	0.774	0.781
XGB	vad	0.769	0.778	0.769	0.778	0.769	0.769	0.769
SVM	vad	0.904	0.815	0.825	0.898	0.825	0.904	0.862
LGBM	vad	0.538	0.907	0.848	0.671	0.848	0.538	0.659

Area under the ROC curve (AUC) with 95% confidence intervals for logistic regression (LR), support vector machine (SVM), decision tree (DT), eXtreme Gradient Boosting (XGB), and LightGBM (LGBM).

### Support vector machine (SVM) as the optimal predictive model and SHAP-based interpretation

**Figure 5:**
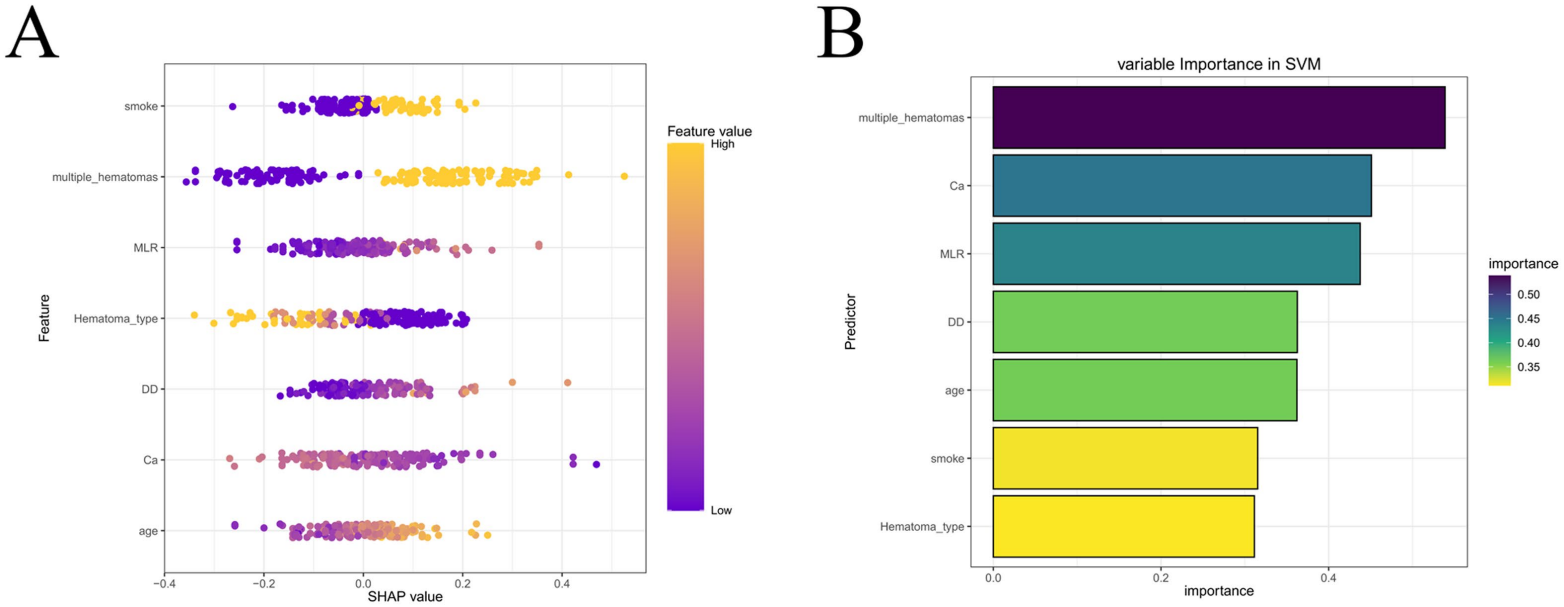
SHAP analysis of the optimal SVM model. **(A)** SHAP summary plot of individual predictors. **(B)** Feature importance ranking. Hematoma type, smoking history, age, D-dimer, MLR, calcium, and multiple hematomas were identified as the most influential predictors of early hematoma progression.

Given the consistent superiority of SVM across multiple evaluation metrics, we selected SVM as the optimal predictive model for early hematoma progression. The SVM classifier achieved robust performance, with a balanced accuracy of 81.2% in the training cohort and 85.9% in the validation cohort. In the training set, the model yielded an overall accuracy of 0.812 (95% CI, 0.758–0.859), sensitivity of 84.8%, specificity of 77.6%, precision of 79.1%, recall of 84.8%, and F1 score of 0.819. Similar results were reproduced in the validation set, where the accuracy was 0.859 (95% CI, 0.777–0.919), sensitivity reached 90.4%, specificity was 81.5%, precision was 82.5%, recall was 90.4%, and the F1 score was 0.862 (Supplementary Figure S6). To further assess the stability and generalizability of the optimal SVM model, we performed a 5-fold cross-validation analysis. As shown in Supplementary Figure S7, the ROC curves were largely consistent across folds, yielding a mean AUC of 0.859 (95% CI, 0.812–0.907) in the training cohort and 0.897 (95% CI, 0.839–0.955) in the validation cohort. These results indicate low performance variability across resampled datasets and support the robustness of the SVM model for potential clinical application. These results underscore the stable discriminative ability of the SVM model, with consistently high sensitivity and specificity, as well as excellent agreement between predicted and observed outcomes across both cohorts. To enhance the interpretability of the SVM model, we incorporated Shapley additive explanations (SHAP), which quantify the contribution of each predictor to the model’s output. As illustrated in Figure 5, hematoma type, smoking history, and age emerged as the strongest contributors, followed by D-dimer, monocyte-to-lymphocyte ratio (MLR), serum calcium, and the presence of multiple hematomas. The SHAP summary plot revealed that higher age, positive smoking history, elevated D-dimer, increased MLR, and the presence of multiple hematomas were positively associated with hematoma progression risk, whereas higher serum calcium was associated with a lower predicted risk of hematoma progression. To facilitate clinically intuitive interpretation beyond global importance ranking, we further provide outcome-stratified SHAP visualizations in the Supplementary Figure S8, including beeswarm plots and representative force/waterfall-style explanations for both progression (positive) and non-progression (negative) samples. These plots clearly display the direction and magnitude of feature contributions and illustrate how key predictors jointly drive individualized predictions toward higher or lower hematoma progression risk. To further enhance clinical interpretability, we additionally provide SHAP dependence plots for the key predictors (Supplementary Figure S9), enabling visual identification of approximate value ranges at which each feature begins to exert a markedly positive contribution to the predicted risk of hematoma progression. In addition, we generated a SHAP heatmap (Supplementary Figure S10) to visualize the instance-level contribution patterns of the final selected predictors in the optimal SVM model. This heatmap provides a patient-centered view of how each feature drives the prediction toward higher or lower risk across individual cases, thereby improving clinical interpretability beyond global importance rankings. The feature importance ranking further emphasized the multifactorial and interdependent nature of hematoma progression, integrating demographic, inflammatory, coagulative, and radiological domains into a coherent risk framework. Together, these results demonstrate that the SVM model not only provides reliable and generalizable predictive performance but also retains interpretability through SHAP analysis, thereby offering a clinically applicable tool for individualized risk stratification and early intervention in patients with traumatic brain injury.

### Nomogram construction and probability-based risk-stratification performance

**Figure 6:**
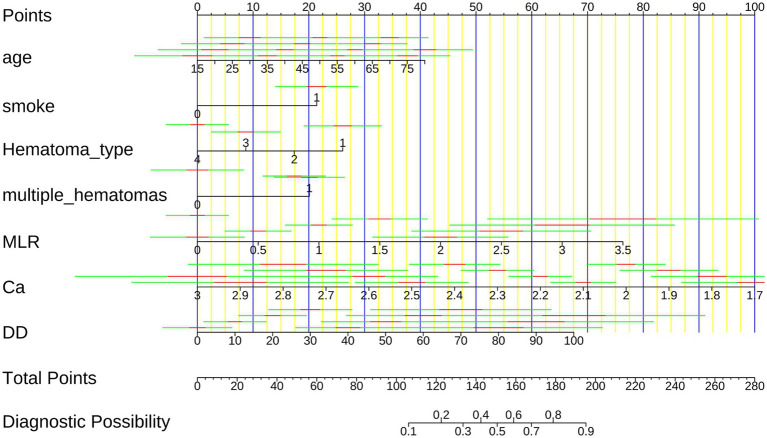
Nomogram for predicting early intracranial hematoma progression. The nomogram integrates hematoma type, smoking history, age, D-dimer, MLR, serum calcium, and multiple hematomas to calculate an individualized risk score. Higher total scores correspond to increased probability of hematoma progression.

Based on the SVM model, we developed a nomogram to provide a clinically applicable tool for individualized prediction of early hematoma progression. As shown in Figure 6, each predictor—hematoma type, smoking history, age, D-dimer, monocyte-to-lymphocyte ratio (MLR), serum calcium, and multiple hematomas—was assigned a weighted score, and the total score corresponded to the estimated probability of progression. This graphical tool enables clinicians to integrate demographic, biochemical, and radiological characteristics into a single quantitative framework for bedside decision-making, with higher scores indicating elevated risk. To further evaluate the predictive performance and clinical utility of the nomogram, we performed probability-based diagnostic analyses. As detailed in Supplementary Table S11, the nomogram demonstrated excellent discriminative ability, with an area under the ROC curve (AUC) consistently above 0.90, sensitivity and specificity greater than 0.80, and favorable positive and negative predictive values, underscoring its reliability in distinguishing patients at risk. Supplementary Figure S11 visualizes the ROC curves of the nomogram compared with individual predictors, showing that the integrated model outperformed any single variable in both the training and validation cohorts. In addition, decision curve analysis (Supplementary Figure S12) demonstrated that the nomogram provided the greatest net clinical benefit across a broad spectrum of threshold probabilities, consistently surpassing individual predictors. Together, these findings establish that the SVM-based nomogram offers not only high discriminative accuracy but also clinically meaningful probability estimates, thereby serving as an interpretable, reliable, and practical decision-support tool for individualized risk stratification of early hematoma progression.

### Risk-stratification performance of the nomoscore

To further examine the risk-stratification performance and clinical interpretability of the SVM-based nomogram, we derived a composite nomoscore and evaluated its association with hematoma progression. As presented in Table 3, increasing quartiles of the nomoscore were strongly correlated with a higher risk of progression. Compared with the lowest quartile (Q1), the odds of hematoma progression increased stepwise across Q2, Q3, and Q4, with the highest quartile showing more than a threefold risk elevation. This dose–response relationship confirmed that the nomoscore provided a rational and discriminative measure of progression risk. Additional stratified analyses are summarized in Supplementary Table S12, which demonstrated consistent associations across clinical subgroups, thereby supporting the robustness of the score. Graphical visualization further reinforced these findings. Supplementary Figure S13 displays a forest plot of odds ratios with 95% confidence intervals for hematoma progression across nomoscore quartiles; a clear monotonic trend was observed, with progressively higher quartiles exhibiting markedly increased risk compared with Q1. Supplementary Figure S14 presents boxplots comparing nomoscore distributions between patients with and without hematoma progression, revealing a significant upward shift in median scores and wider interquartile ranges among patients who developed progression. These visualizations complement the tabulated results, underscoring that the nomoscore effectively stratifies patients into distinct risk categories with strong discriminatory power. Together, these findings validate the nomoscore as a clinically meaningful extension of the SVM-based model, translating statistical prediction into an interpretable scoring system with both robustness and bedside applicability.

**Table 3:** Distribution of hematoma progression risk across quartiles of the nomoscore.

Name	Desc	0 (*N* = 125)	1 (*N* = 125)	OR (multivariable)
NomoScore	Q1	58 (46.4%)	5 (4%)	
	Q2	43 (34.4%)	19 (15.2%)	5.13 (1.77–14.81, *p* = 0.002)
	Q3	16 (12.8%)	46 (36.8%)	33.35 (11.37–97.83, *p* < 0.001)
	Q4	8 (6.4%)	55 (44%)	79.75 (24.59–258.67, *p* < 0.001)

Odds ratios (ORs) with 95% confidence intervals (CIs) are shown for each quartile relative to the reference group (Q1).

### Sensitivity analyses

**Figure 7:**
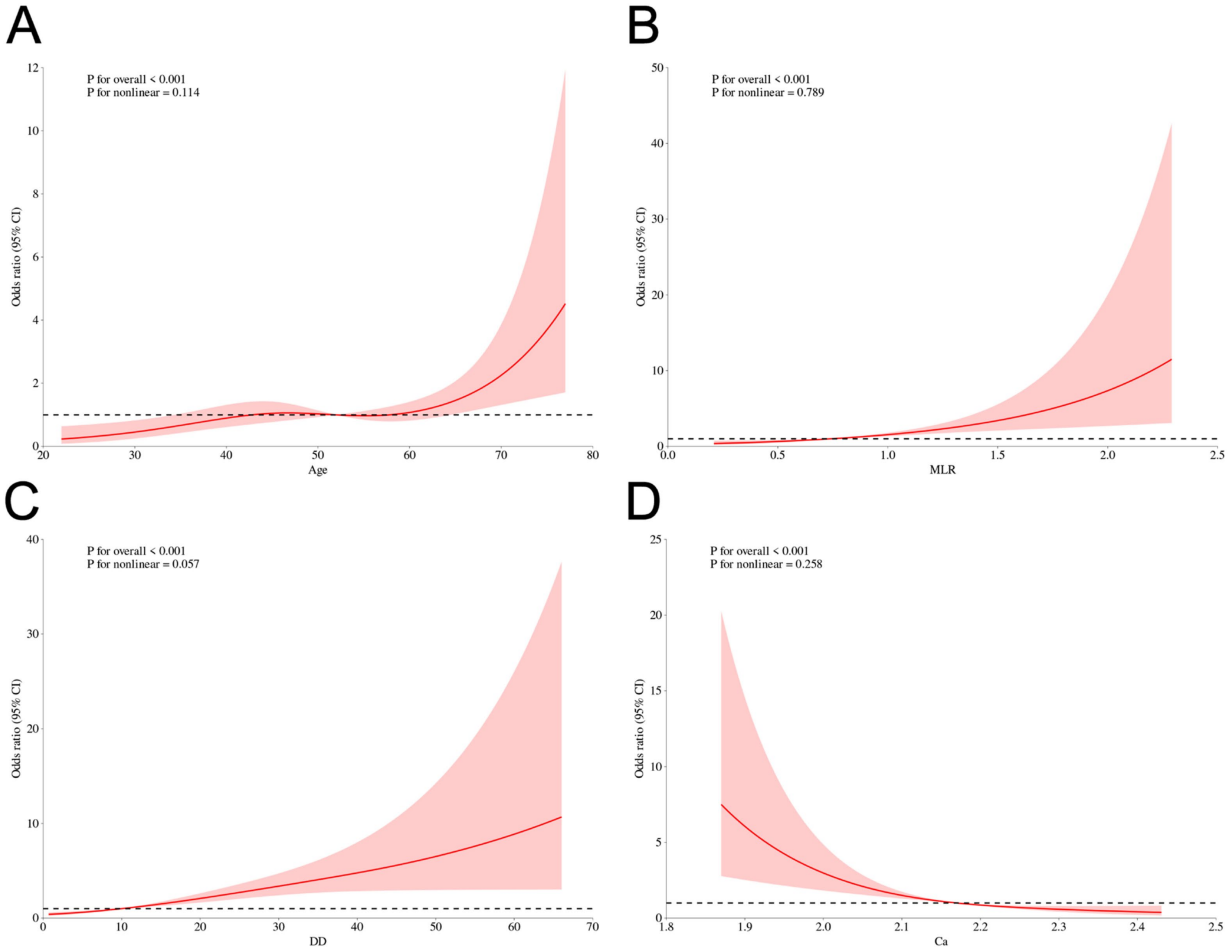
Sensitivity analysis using restricted cubic spline (RCS) curves for continuous predictors and early hematoma progression. RCS plots for continuous predictors and early hematoma progression: **(A)** Age, **(B)** MLR, **(C)** D-dimer, and **(D)** Calcium. Solid line, odds ratio (OR); shaded area, 95% CI; dashed line, OR = 1. *p* values for overall association and nonlinearity are shown.

To further characterize the relationships between the final selected continuous predictors and early hematoma progression, we performed restricted cubic spline (RCS) analyses as a sensitivity analysis. As shown in Figure 7, age (Figure 7A), MLR (Figure 7B), and D-dimer (Figure 7C) exhibited overall positive associations with progression risk, whereas serum calcium showed an inverse association (Figure 7D). Importantly, the spline curves provide an intuitive depiction of potential nonlinear patterns across the observed ranges, complementing the machine-learning findings by illustrating how risk changes continuously with each biomarker rather than assuming strict linear effects. In addition, we performed subgroup analyses to examine whether the associations of smoking history and multiple hematomas with early hematoma progression were consistent across clinically relevant strata. As shown in Supplementary Figure S15, both factors remained significantly associated with progression risk across most subgroups, and no statistically significant effect modification was observed in the majority of stratifications based on demographic characteristics, comorbidities, injury severity, and radiological features (P for interaction generally > 0.05). These findings support the robustness of smoking history and multiple hematomas as risk indicators and suggest that their effects are broadly stable across patient subpopulations.

## Discussion

To date, few studies have developed machine learning (ML) models specifically designed to predict early intracranial hematoma progression in traumatic brain injury (TBI) patients. In this study, we proposed and validated an interpretable ML-based model to address this important clinical challenge. Our main findings can be summarized as follows: (1) the support vector machine (SVM) algorithm exhibited the best predictive performance, achieving an AUC of 0.925 in the validation cohort; (2) SHapley Additive exPlanations (SHAP) identified hematoma type, smoking history, age, D-dimer, monocyte-to-lymphocyte ratio (MLR), serum calcium, and multiple hematomas as the most influential predictors; (3) the model’s nomogram and nomoscore demonstrated clinical interpretability, providing practical bedside decision support. Compared with prior seminal ML studies in TBI that largely focused on broader endpoints (e.g., mortality or long-term functional outcome) and used different outcome definitions/time windows and feature sets, our strict 24-h progression endpoint and multimodal predictors may partly explain differences in reported performance metrics and in which algorithm emerges as optimal across studies (20, 21).

We constructed multiple ML models using seven key variables selected by LASSO regression from a broad set of clinical features. Among the algorithms tested, including logistic regression, decision tree, SVM, eXtreme Gradient Boosting (XGB), and LightGBM, the SVM model achieved the most robust balance between discrimination and calibration. Its kernel-based framework is particularly advantageous in capturing nonlinear interactions between risk factors, thereby avoiding the limitations of conventional regression models (22–24). Importantly, probability-based performance evaluation and nomoscore validation confirmed the stability of this model, reinforcing its translational value in real-world clinical settings (25).

Our model identified age as one of the strongest predictors of hematoma progression. This aligns with previous evidence that older patients exhibit impaired cerebrovascular autoregulation, increased blood–brain barrier (BBB) fragility, and reduced capacity for hemostatic repair (26, 27). For example, Roozenbeek et al. showed that TBI patients aged over 60 had significantly higher risks of intracranial lesion enlargement compared with younger counterparts (28). In addition, advanced age is frequently associated with comorbidities such as hypertension or atrial fibrillation, often requiring antiplatelet or anticoagulant therapy, further destabilizing hemostasis (29). These cumulative vulnerabilities likely explain the consistent association of age with hematoma expansion.

Smoking history was another important factor highlighted by SHAP. Cigarette smoke promotes chronic endothelial injury, platelet activation, and oxidative stress, all of which contribute to coagulopathy (30). Experimental studies have demonstrated that nicotine enhances thrombin generation while impairing fibrinolytic capacity, thereby creating unstable clots (31, 32). Clinically, Sivandzade F, et al. reported that smokers with TBI had a significantly higher rate of delayed intracranial hemorrhage compared with non-smokers, independent of injury severity (33, 34). Furthermore, smoking-induced systemic inflammation has been linked to dysregulated leukocyte activity and cytokine release, which destabilize clot formation and worsen hematoma progression (35).

Radiological features such as hematoma type and multiple hematomas were also decisive predictors. Among hematoma subtypes, intraparenchymal hemorrhage carries the highest risk of progression, likely due to the rupture of fragile penetrating arterioles and poor tamponade effect (36). Previous studies have shown that intraparenchymal hematomas are more prone to secondary expansion compared with epidural or subdural hematomas (37). In our cohort, patients with multiple hematomas were at particularly high risk, consistent with findings from Narayan et al., who demonstrated that poly-lesional injuries are associated with diffuse axonal and vascular disruption, elevated intracranial pressure, and higher mortality (38). Multiple lesions also reflect more severe biomechanical impact, which amplifies systemic coagulopathy and inflammatory cascades (39).

Laboratory biomarkers provided further mechanistic insights. D-dimer was strongly associated with hematoma progression. Elevated D-dimer reflects active fibrinolysis and systemic coagulation activation, conditions frequently observed after severe TBI (40). Previous work by Gando et al. demonstrated that TBI patients with markedly elevated D-dimer levels had higher risks of disseminated intravascular coagulation (DIC) and secondary bleeding (41). Similarly, Chhabra et al. confirmed that admission D-dimer independently predicted hematoma enlargement in acute head trauma (42). Thus, D-dimer serves as both a marker of clot instability and a surrogate indicator of systemic hemostatic dysregulation.

The monocyte-to-lymphocyte ratio (MLR) was another critical predictor. Monocytes infiltrate injured brain tissue, releasing pro-inflammatory mediators such as IL-1β, TNF-*α*, and IL-6, which increase BBB permeability (43). Conversely, post-traumatic lymphopenia reflects immune suppression, a well-described phenomenon after severe injury (44). Together, an elevated MLR reflects the imbalance of hyperinflammation and immunosuppression. Zhang et al. demonstrated that elevated MLR independently predicted poor neurological outcomes in intracerebral hemorrhage patients (45), while Chen et al. reported similar associations in severe trauma (46). In this context, MLR represents a dynamic biomarker linking systemic inflammation and cerebral vascular fragility.

Serum calcium showed an inverse association with early hematoma progression in our cohort, which is consistent with its established role in hemostasis; therefore, we interpret it primarily as a readily available biomarker that contributes to early risk stratification rather than as a novel mechanistic discovery. Hypocalcemia impairs platelet aggregation and reduces the activity of key coagulation factors (II, VII, IX, X), thereby predisposing to uncontrolled bleeding (47). In addition, hypocalcemia has been linked to endothelial dysfunction and worse outcomes in critically ill patients (48). A study by Xu et al. showed that low calcium levels on admission were significantly associated with hematoma expansion in spontaneous intracerebral hemorrhage (49). Our findings extend this evidence to TBI, underscoring the role of calcium homeostasis in clot stability and hematoma control.

Finally, multiple hematomas deserve emphasis as an integrated risk signal. Beyond their direct mechanical impact, multiple lesions often trigger a cascade of systemic responses, including disseminated coagulopathy, increased ICP, and global cerebral hypoperfusion (50). Prior work by Maas et al. highlighted that patients with multiple intracranial lesions not only face higher risks of progression but also demonstrate worse long-term functional outcomes (51, 52). In combination with systemic inflammation and coagulopathy, this may explain why multiple hematomas emerged as one of the most decisive predictors in our model.

We agree that the core ML components (model families, internal validation strategies, and performance metrics) are now widely used in the TBI literature, and that interpretability tools such as SHAP and nomogram-style clinical translation have also been reported. For example, Wei et al. applied an ML workflow with SHAP-based interpretation in a pediatric TBI prognostic setting, reflecting the broader trend toward explainable ML rather than ‘black-box’ reporting. Importantly, the fact that prior studies have identified different ‘best-performing’ algorithms does not indicate inconsistency, but rather underscores the heterogeneity of TBI cohorts and study designs (53). Logistic regression may remain competitive in some datasets with relatively linear structure and limited feature interactions (e.g., Tu et al.) (54), whereas tree-boosting methods may excel in others with stronger nonlinearities (e.g., Wang et al.) (55). Similarly, SVM has shown strong performance in certain registry-based TBI outcome prediction tasks (e.g., Abujaber et al.) (56). Differences across studies commonly arise from variations in endpoint definition and time horizon, population severity and case-mix, feature availability (especially imaging-derived variables), sample size and class imbalance, preprocessing/imputation choices, and hyperparameter tuning/validation protocols. In this context, our rationale was to compare multiple established algorithms under a unified feature set and evaluation framework, and to select the model demonstrating the most robust overall performance for the clinically actionable endpoint of early hematoma progression.

Taken together, these predictors illustrate the multifactorial nature of hematoma progression, driven by interactions among demographic variables, lifestyle factors, imaging features, and laboratory biomarkers. The convergence of vascular dysfunction, systemic inflammation, coagulation imbalance, and metabolic disturbance provides a coherent mechanistic framework, explaining both the clinical heterogeneity of TBI and the predictive validity of our model (57, 58).

In the present work, the added value lies not in ‘discovering’ entirely unknown risk factors, but in quantifying and operationalizing multifactorial risk for an actionable, early endpoint—early intracranial hematoma progression—which remains clinically important yet relatively underexplored. Specifically, ML enables the integration of heterogeneous domains, including radiological subtype and multiplicity as well as coagulation, inflammation, and metabolic markers, and can capture nonlinear contributions that are not easily expressed by linear assumptions. Methodologically, we applied rigorous feature selection and conducted a systematic head-to-head comparison of five ML algorithms, identifying SVM as the most robust model. To further strengthen interpretability and clinical relevance, we complemented performance evaluation beyond discrimination by assessing calibration and decision-analytic net benefit, and we used SHAP to link model predictions with clinically and biologically plausible patterns. Finally, by translating the optimal model into a clinically oriented nomogram and nomoscore-based risk stratification scheme, we bridge computational analytics with bedside decision-making and provide a transparent framework that may help guide early, targeted monitoring and interventions.

Specifically, the SVM model can capture nonlinear contributions and joint patterns that are not easily expressed under linear assumptions, while SHAP-based explanations help visualize how predictors jointly push an individual prediction toward higher or lower risk. In this way, the model moves beyond confirming ‘known associations’ to delivering an interpretable, bedside-oriented tool for actionable early risk stratification. In routine practice, experienced clinicians may recognize these risk factors, but integrating them simultaneously into a consistent, patient-specific probability estimate can be subjective and may vary across providers. Therefore, our nomogram/nomoscore is intended to augment—not replace—clinical judgment by offering an objective and reproducible risk estimate, and the decision-curve analysis suggests potential clinical utility across a range of decision thresholds. Nevertheless, several limitations should be noted. First, although a broad set of clinical and laboratory features was included, missing data inherent to retrospective cohorts may have introduced bias. Second, as the study was based on a single-center population in China, external generalizability remains to be tested in different ethnic and healthcare contexts. Third, residual confounding is inevitable in observational research, despite statistical adjustment. Finally, although the model performed well in internal validation, a direct head-to-head comparison with physician assessment and large-scale multicenter prospective implementation studies are needed before clinical adoption.

Future work is warranted to further strengthen clinical translation. Although SHAP enables sample-level explanations, the present work focused on model development and retrospective validation. In future studies, we will incorporate individual-level longitudinal trajectories (e.g., dynamic changes in imaging and laboratory parameters over time) to examine how evolving patient states influence predicted hematoma progression risk. We also plan prospective, multicenter validation and implementation studies to evaluate the real-world clinical impact of model-assisted decision-making. f externally and prospectively validated, the model could be deployed as an admission-time risk calculator (e.g., EHR-integrated) to support early risk stratification and guide individualized decisions on repeat CT timing and monitoring intensity, particularly by prioritizing earlier imaging and closer surveillance for high-risk patients.

## Conclusion

In this study, we developed and validated an interpretable and high-performing machine learning model—support vector machine (SVM)—to predict early intracranial hematoma progression in traumatic brain injury (TBI) patients. The model demonstrated excellent discrimination and calibration, with strong clinical interpretability enabled by SHAP analysis and nomogram construction. By integrating key demographic, radiological, and laboratory features, this model provides a reliable and practical tool for early risk stratification. Its translational potential lies in optimizing individualized management strategies and guiding timely intervention, thereby improving outcomes in this vulnerable population.

## Data Availability

The raw data supporting the conclusions of this article will be made available by the authors, without undue reservation.
