# Supplementary material for: Explainable machine learning reveals multifactorial drivers of early intracranial hematoma progression in traumatic brain injury: development of a SHAP-guided SVM nomogram
Source: Front Neurol. 2026 Feb 5;17:1718794. doi: 10.3389/fneur.2026.1718794 (PMC12916409; doi:10.3389/fneur.2026.1718794)
Supplement: Supplementary file 1 [file Table_1.docx]

**
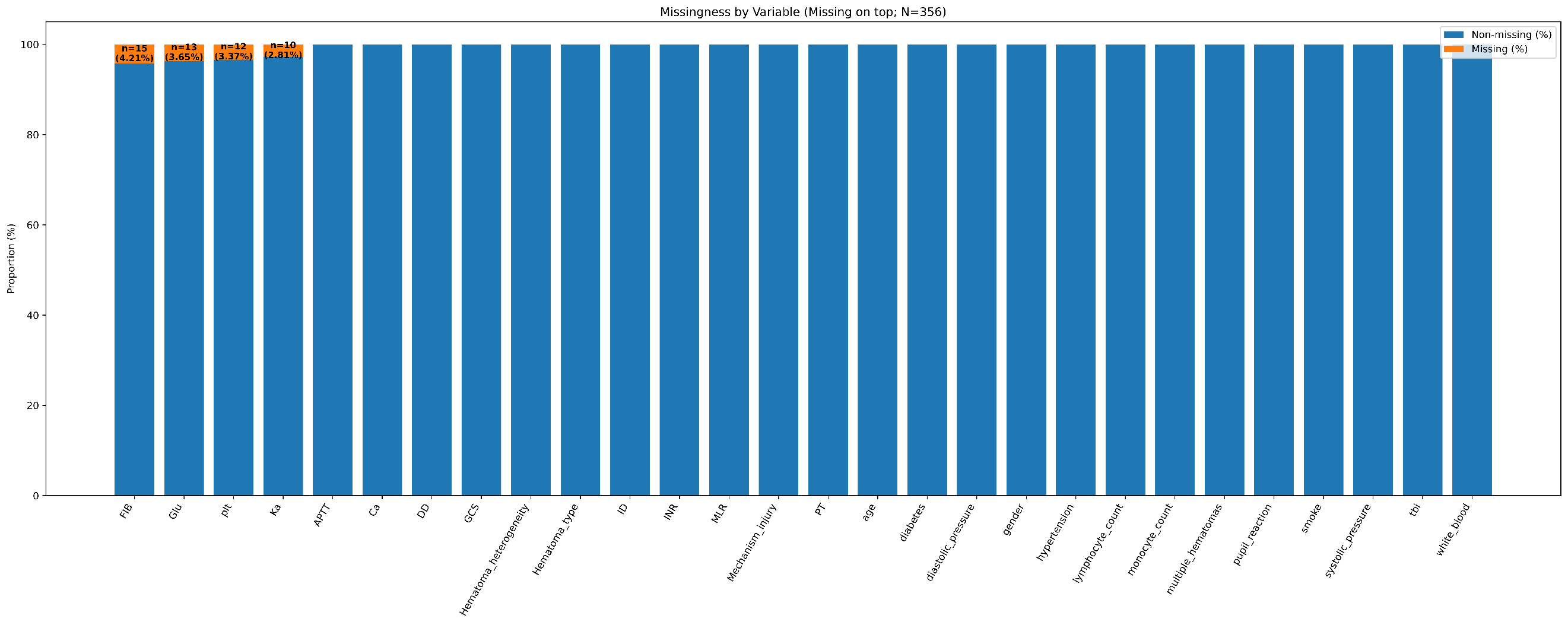
**

***Figure S1. Missing Data Rate by Variable.*** *Proportion of missing data for each variable in the dataset.*

**
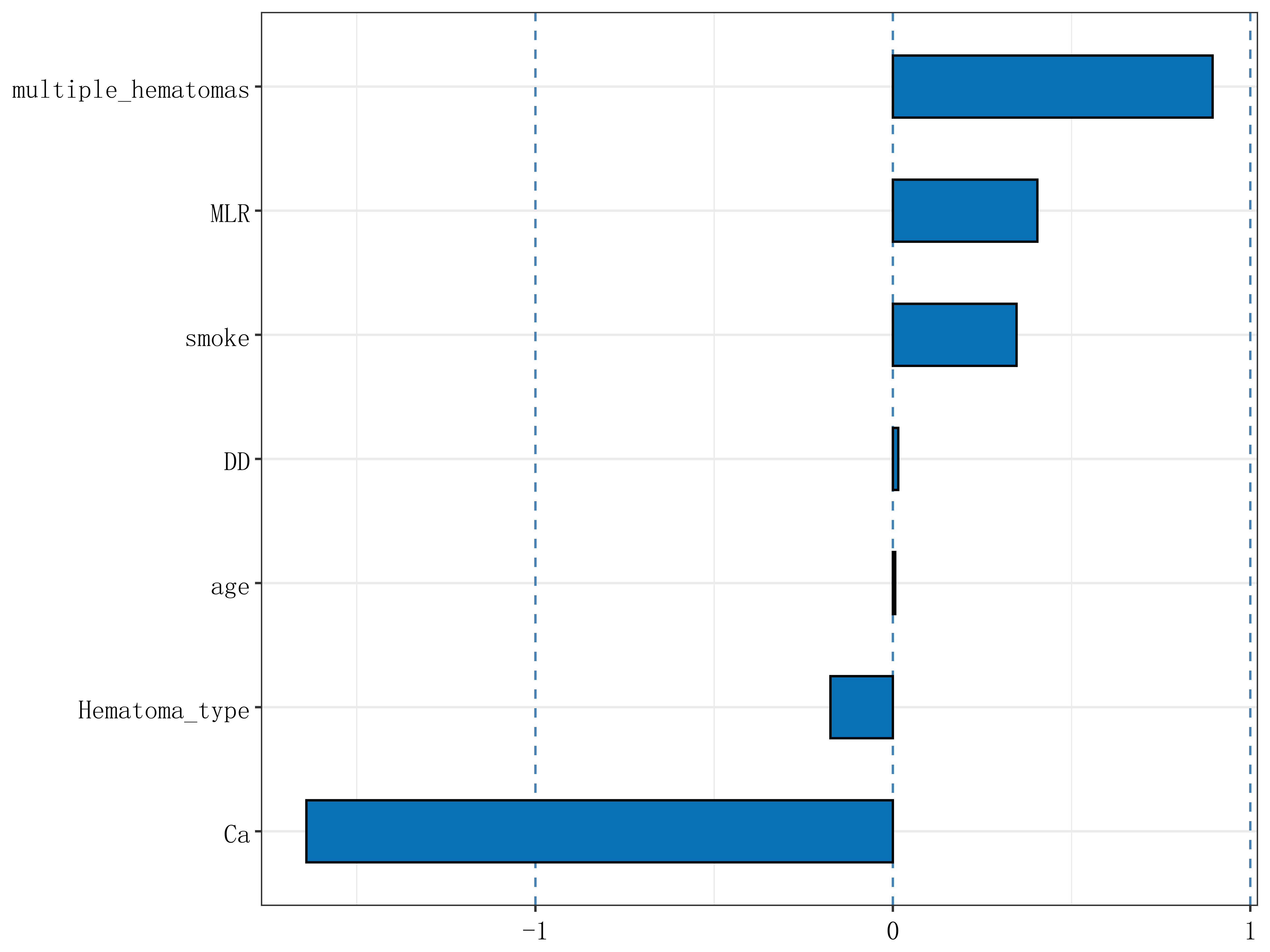
**

***Figure S2. Cross-validation plot of LASSO regression.***

*Deviance curves across log(λ) from 10-fold cross-validation. Lambda.1se was selected to balance model sparsity and performance.*

*
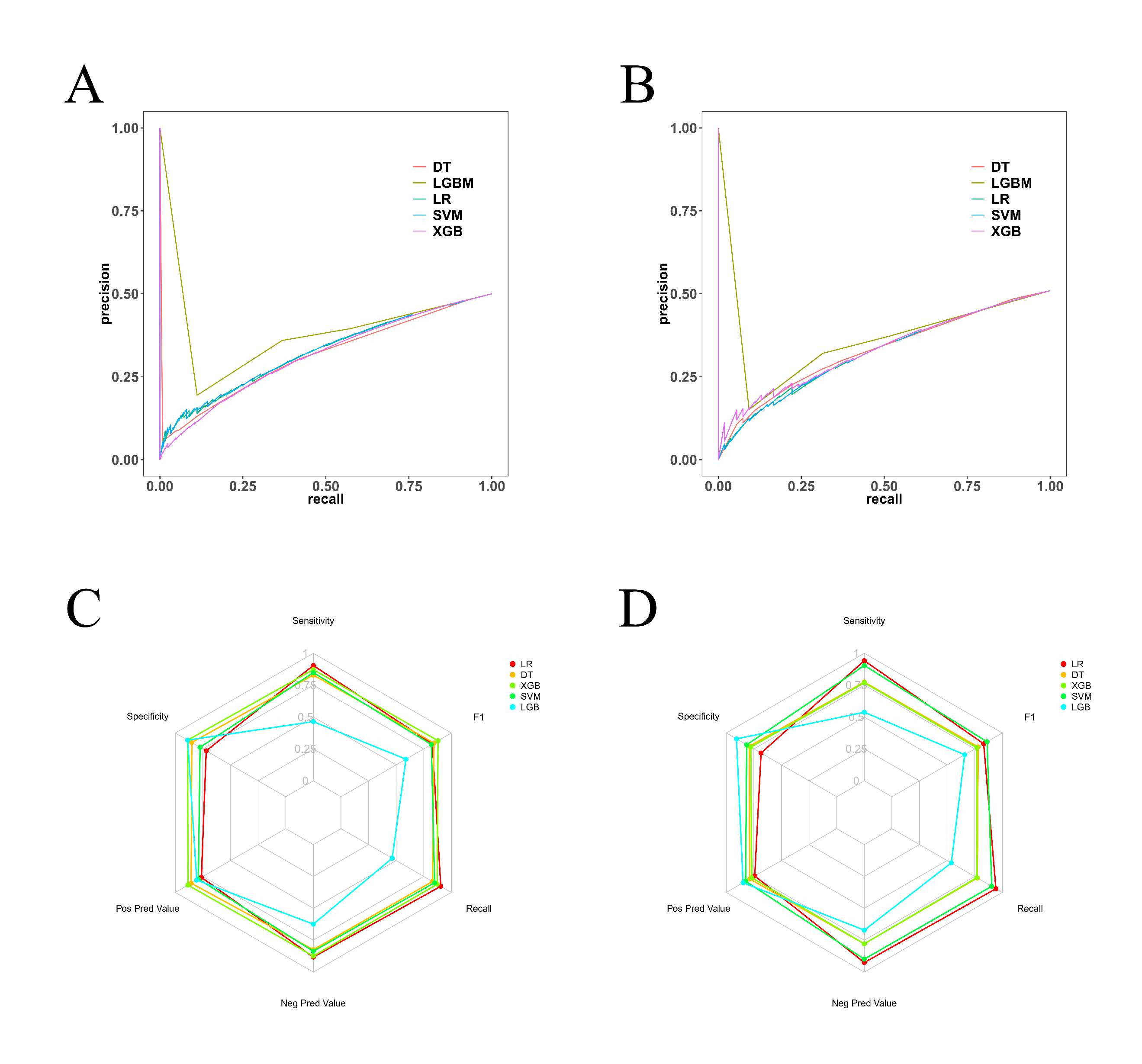
*

***Figure S3. Precision–recall (PR) curves and related parameters of five machine-learning models. (A)*** *PR curves of the five models in the training cohort.****(B)*** *PR curves of the five models in the validation cohort.****(C)*** *PR-based performance metrics (sensitivity, specificity, precision, recall, F1 score) in the training cohort.****(D)*** *PR-based performance metrics in the validation cohort.*

*
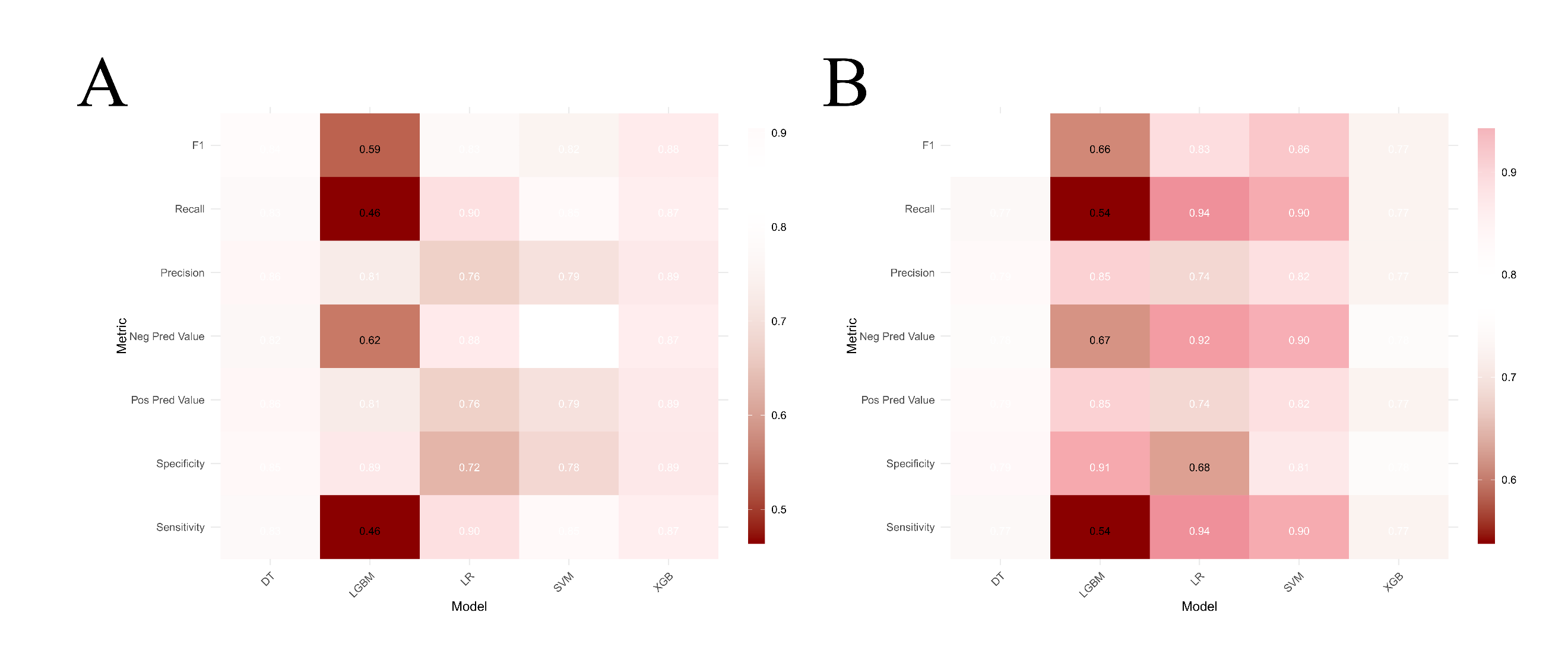
*

***Figure S4. Confusion-matrix–based performance metrics of five machine-learning models.***

***(A)*** *Heatmap of classification metrics in the training cohort.****(B)*** *Heatmap of classification metrics in the validation cohort. Metrics include sensitivity, specificity, positive predictive value, negative predictive value, precision, recall, and F1 score.*

*
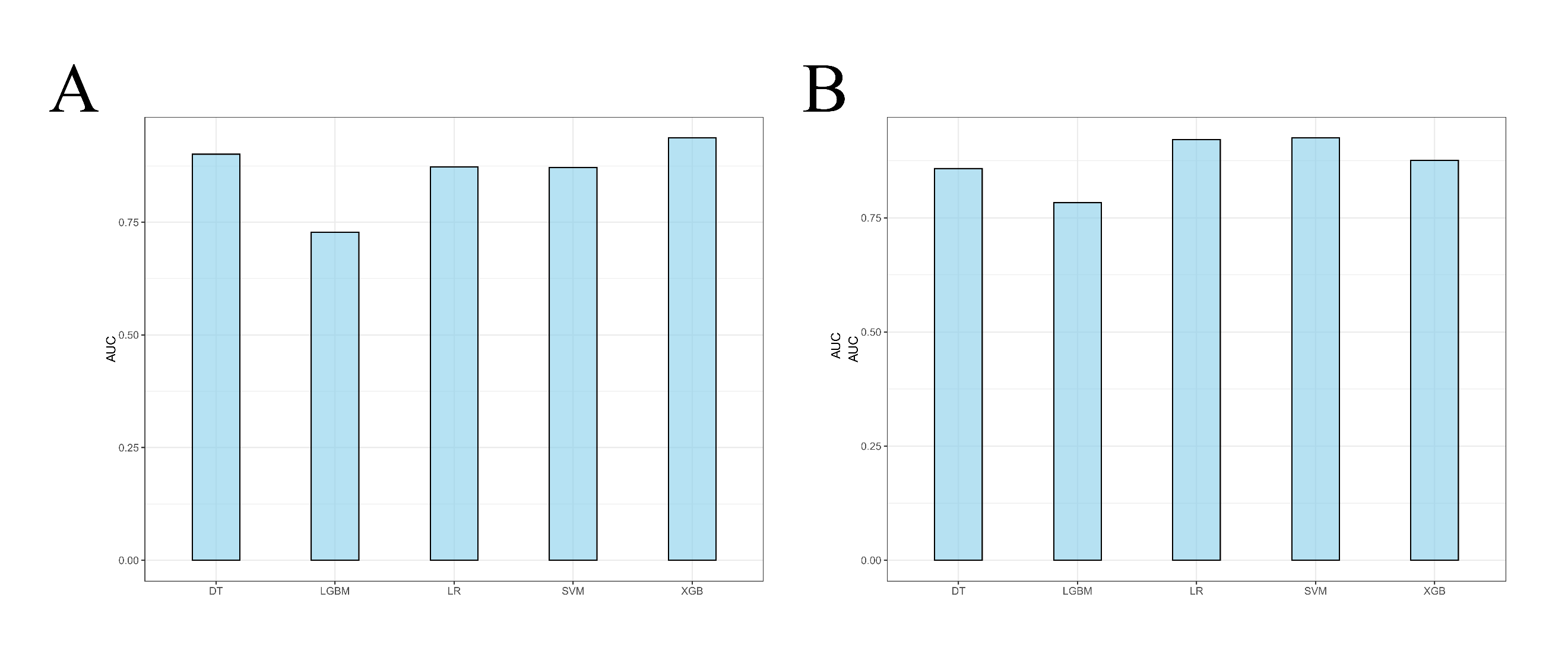
*

***Figure S5. Comparative AUC distributions of five machine-learning models.***

***(A)*** *Boxplots of AUC values in the training cohort.****(B)*** *Boxplots of AUC values in the validation cohort.*


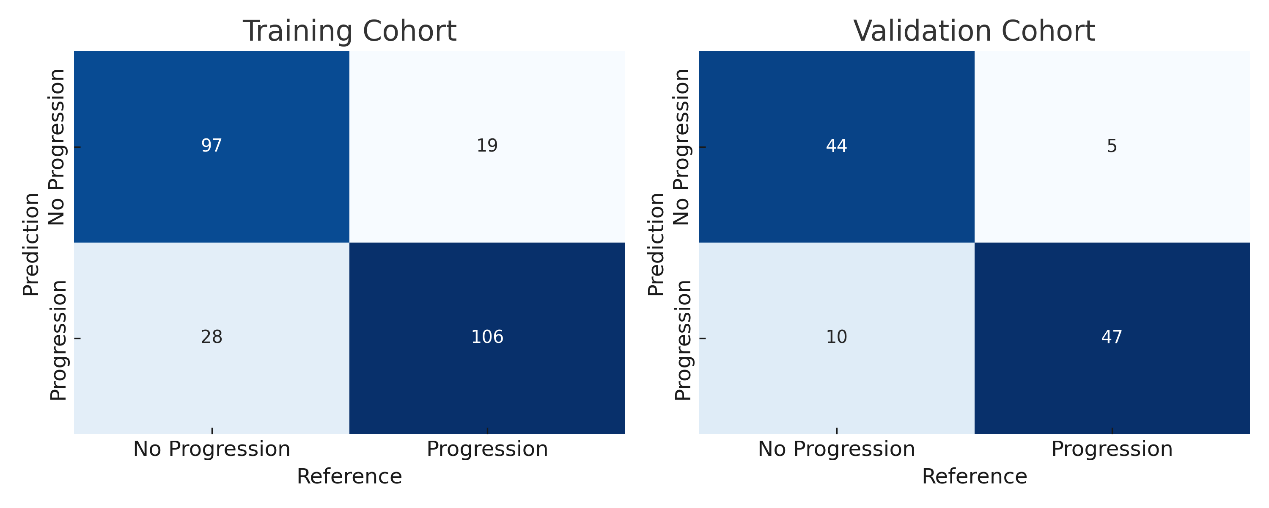


***Figure S6. Confusion matrices of the SVM model in the training and validation cohorts.***

*Confusion matrices of the SVM model in the training (left) and validation (right) cohorts. Rows show predicted classes and columns show reference classes. Diagonal cells indicate correct predictions; off-diagonal cells indicate errors. Numbers denote patient counts.*

***
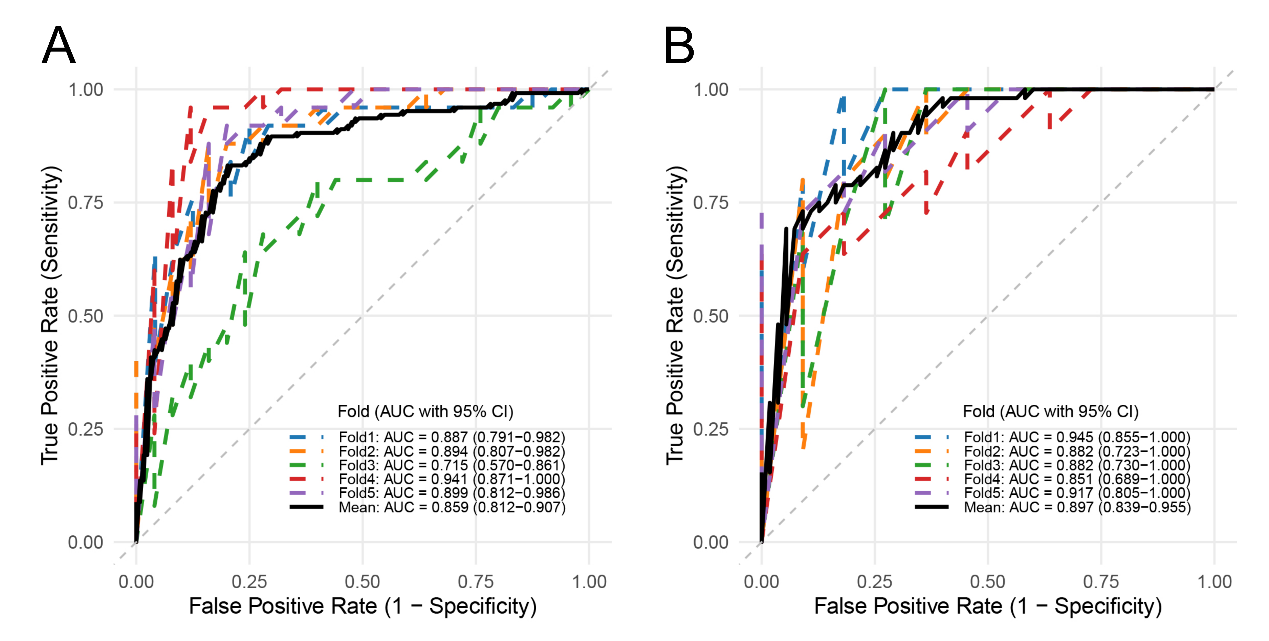
***

***Figure S7 . Five-fold cross-validation ROC curves of the optimal SVM model.***

*Receiver operating characteristic (ROC) curves from 5-fold cross-validation of the SVM model in the training cohort (A) and validation cohort (B). Dashed lines denote fold-specific ROC curves (AUC with 95% CI), the solid black line indicates the mean ROC, and the grey diagonal represents no discrimination.*

*
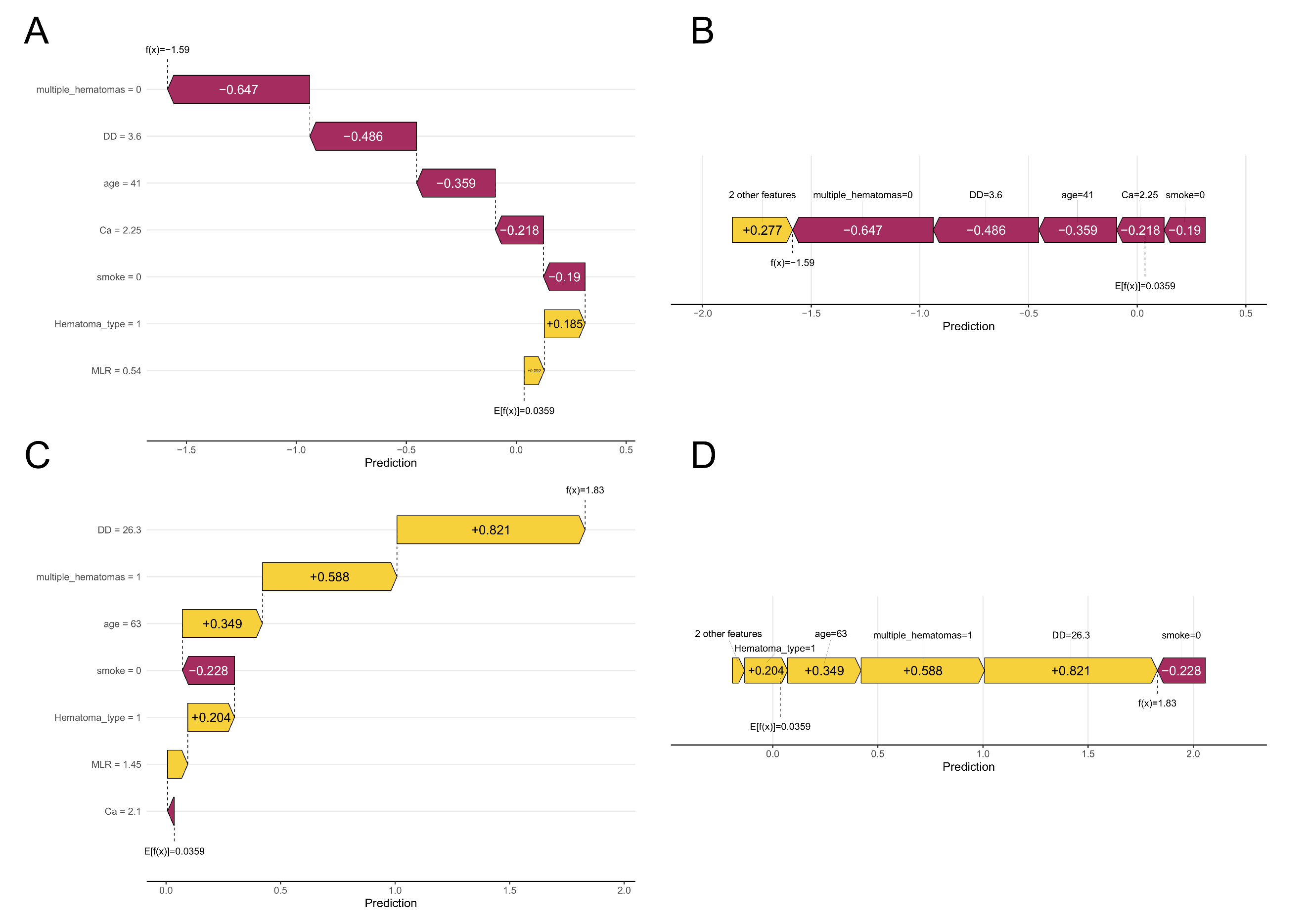
*

***Figure S8. Waterfall and Force Plots for Negative Samples.***

***(A)*** *Waterfall plot showing individual feature contributions for negative samples.****(B)*** *Force plot illustrating the combined effect of features on predictions for negative samples.****(C)*** *Waterfall plot for a different set of negative samples.****(D)*** *Force plot for the same negative samples.*

***
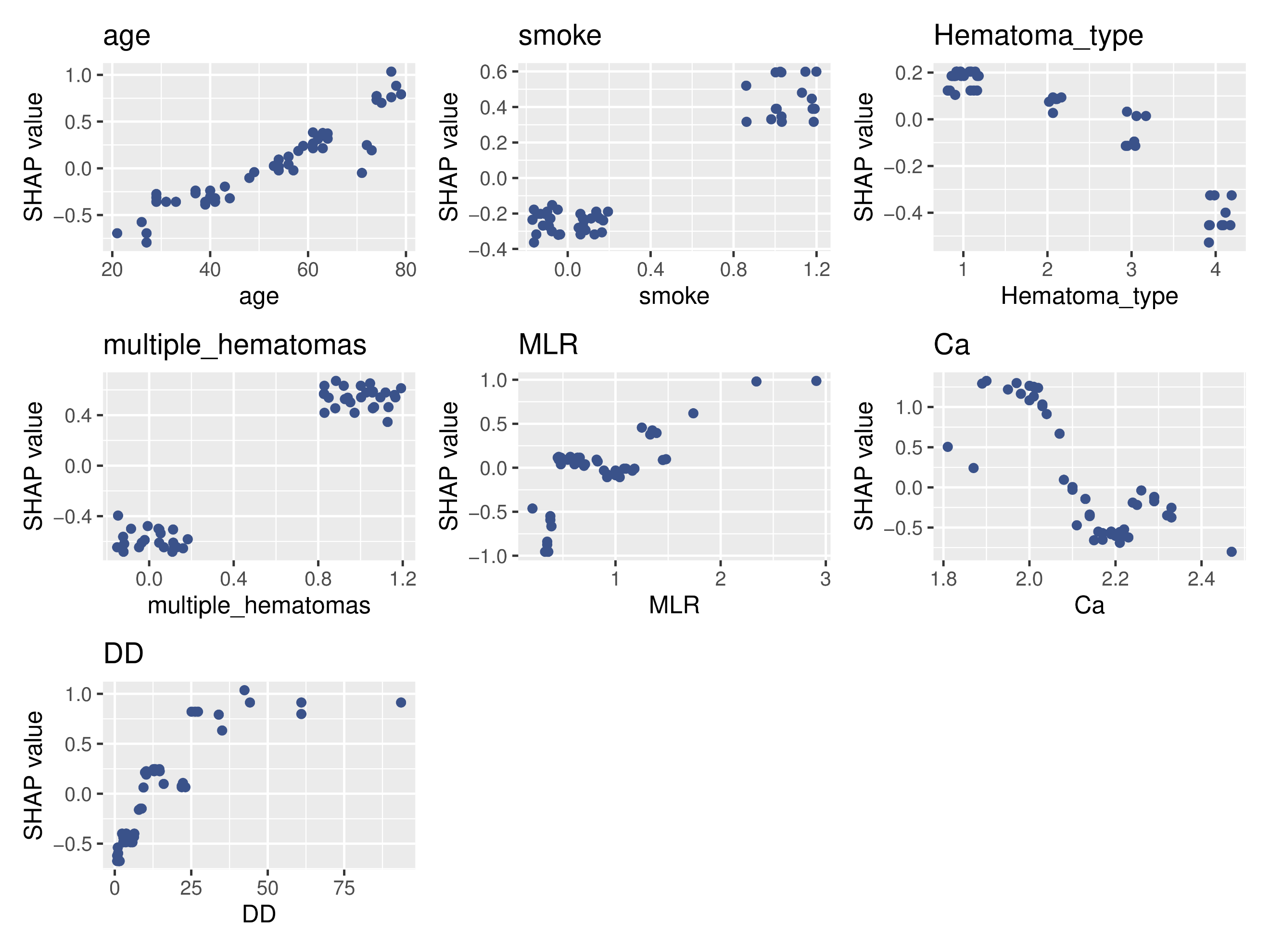
***

***Figure S9. SHAP Dependence Plots for Key Predictors.*** *The plots show the relationship between each key predictor and its corresponding SHAP value. Each point represents an individual sample.*


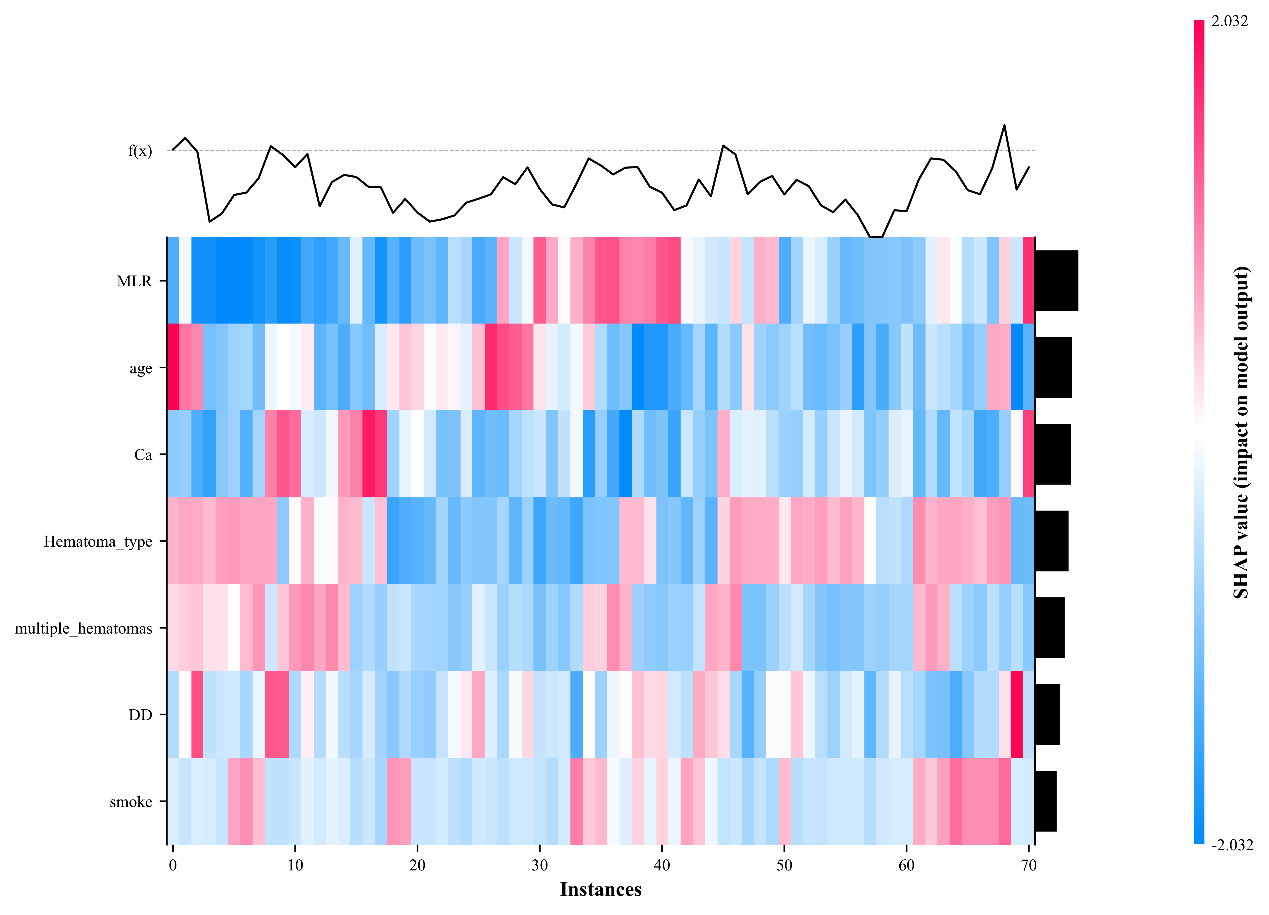


***Figure S10*** ***. SHAP heatmap showing instance-level feature contributions of the optimal SVM model for early hematoma progression.*** *Columns denote individual patients and rows denote the final selected predictors. Colors represent SHAP values (red: increased predicted risk; blue: decreased predicted risk). The top curve shows the corresponding model output* $f(x)$*, and the right-side bars summarize overall feature importance (mean absolute SHAP value).*


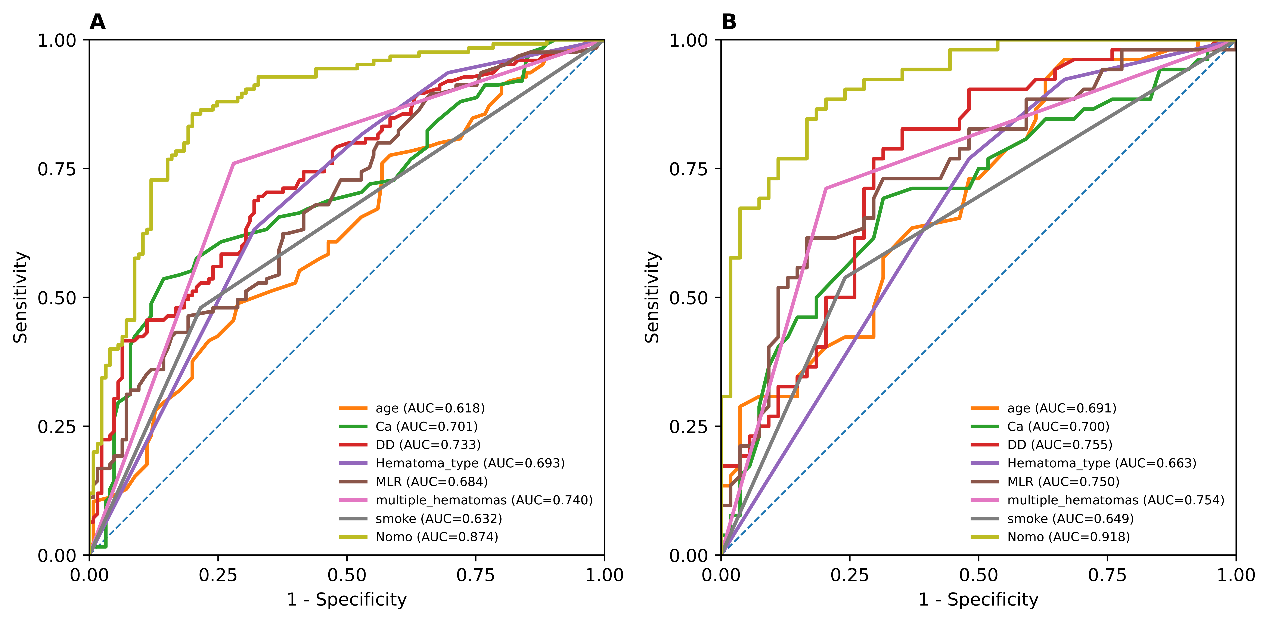


***Figure S11. ROC curves of the nomogram and individual predictors in the training and validation cohorts.***
*(A) ROC analysis in the training cohort. (B) ROC analysis in the validation cohort. The nomogram achieved higher AUC values than any single predictor, demonstrating superior discriminative performance across both cohorts.*

*
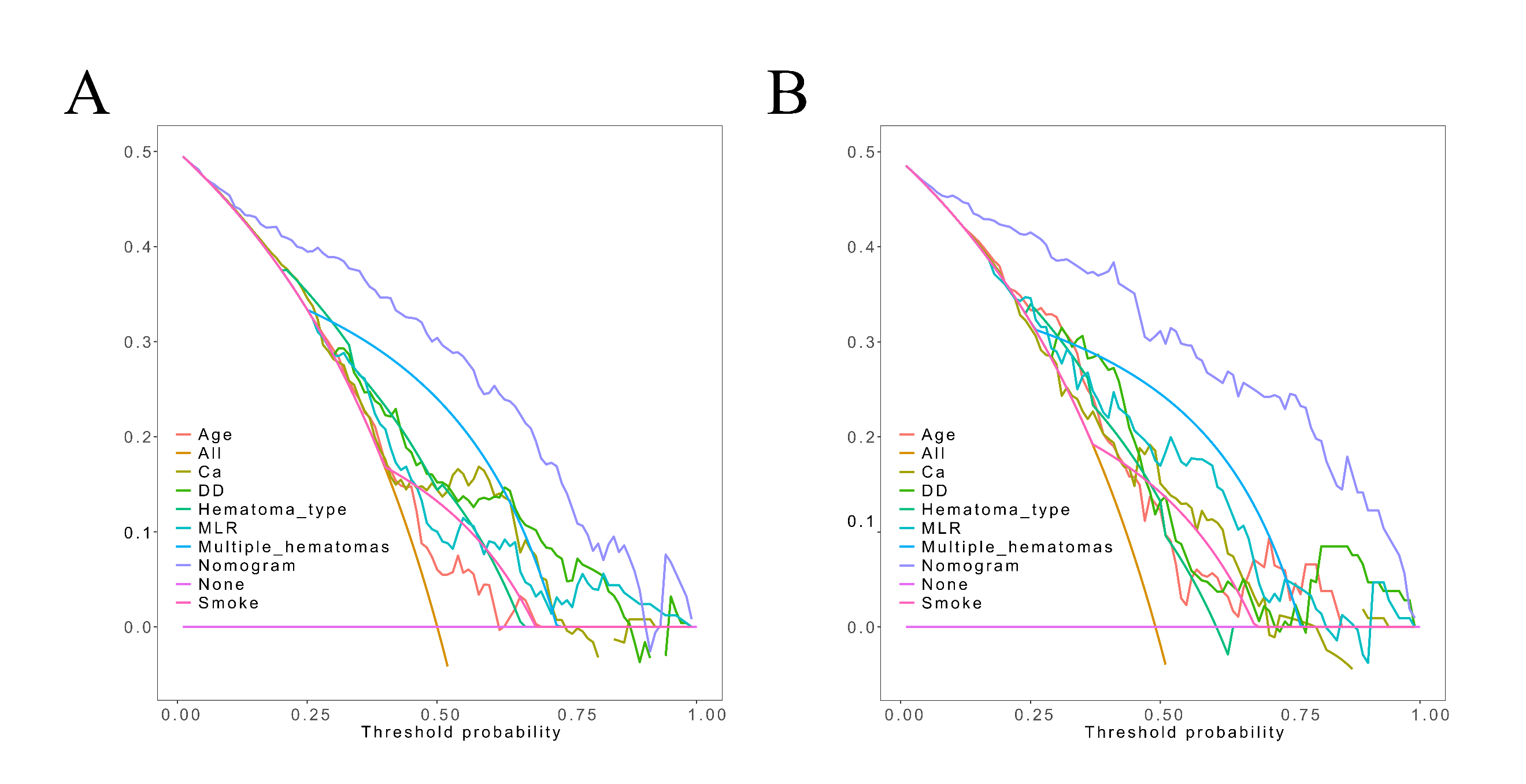
*

***Figure S12. Decision curve analysis (DCA) of the nomogram and individual predictors.*** *(A) Training cohort. (B) Validation cohort. The nomogram yielded the greatest net clinical benefit across a wide range of threshold probabilities, consistently outperforming individual predictors and confirming its clinical utility.*

*

*

***Figure S13. Odds ratios of hematoma progression across nomoscore quartiles.***

*Forest plot showing odds ratios (ORs) with 95% confidence intervals (CIs) for hematoma progression across quartiles of the nomoscore. A stepwise increase in risk was observed from Q1 to Q4, indicating strong monotonic association between higher scores and progression risk.*

*
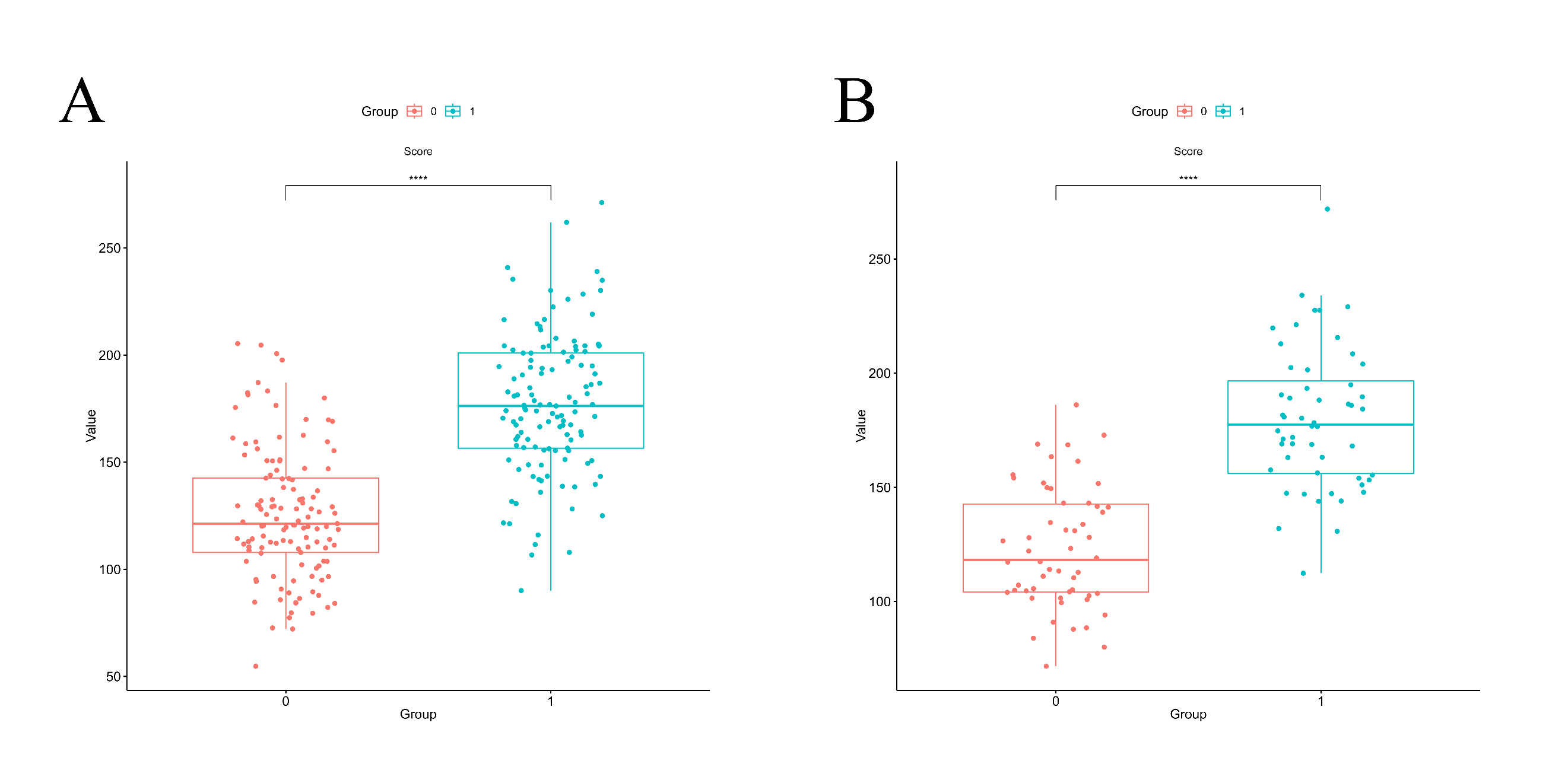
*

***Figure S14. Distribution of nomoscores in patients with and without hematoma progression.***

*Boxplots comparing nomoscore values between patients who developed hematoma progression and those who did not. Patients with progression consistently exhibited significantly higher scores, supporting the discriminative capacity of the nomoscore.*





***Figure S15:*** ***Subgroup analyses of the associations between multiple hematomas (A) and smoking history (B) and early hematoma progression.***

*Forest plots showing subgroup-specific odds ratios (ORs) with 95% confidence intervals for early hematoma progression according to multiple hematomas (A) and smoking history (B). P values for interaction are provided to assess effect modification across subgroups.*
