# Supplementary material for: Explainable machine learning reveals multifactorial drivers of early intracranial hematoma progression in traumatic brain injury: development of a SHAP-guided SVM nomogram
Source: Front Neurol. 2026 Feb 5;17:1718794. doi: 10.3389/fneur.2026.1718794 (PMC12916409; doi:10.3389/fneur.2026.1718794)
Supplement: Supplementary file 2 [file Table_2.docx]

Table S1**.** Comparison of baseline clinical and laboratory characteristics between patients with and without early intracranial hematoma progression.

|  | [ALL] N=356 | 0 N=179 | 1 N=177 | p.overall |
| --- | --- | --- | --- | --- |
| Gender: |  |  |  | 0.084 |
| Male | 277 (77.81%) | 132 (73.74%) | 145 (81.92%) |  |
| Female | 79 (22.19%) | 47 (26.26%) | 32 (18.08%) |  |
| Age | 52.00 [41.00;62.25] | 49.00 [37.00;57.00] | 55.00 [46.00;66.00] | <0.001 |
| Hypertension: |  |  |  | 0.095 |
| No | 270 (75.84%) | 143 (79.89%) | 127 (71.75%) |  |
| Yes | 86 (24.16%) | 36 (20.11%) | 50 (28.25%) |  |
| Diabetes: |  |  |  | 0.022 |
| No | 304 (85.39%) | 161 (89.94%) | 143 (80.79%) |  |
| Yes | 52 (14.61%) | 18 (10.06%) | 34 (19.21%) |  |
| Smoke: |  |  |  | <0.001 |
| No | 228 (64.04%) | 139 (77.65%) | 89 (50.28%) |  |
| Yes | 128 (35.96%) | 40 (22.35%) | 88 (49.72%) |  |
| Systolic_pressure | 123.00 [120.00;138.00] | 120.00 [120.00;130.00] | 130.00 [120.00;140.00] | 0.006 |
| Diastolic_pressure | 80.00 [72.00;89.00] | 80.00 [70.00;84.00] | 80.00 [75.00;89.00] | 0.095 |
| Mechanism_of_injury: |  |  |  | 0.843 |
| Traffic accident | 105 (29.49%) | 51 (28.49%) | 54 (30.51%) |  |
| Fall from height | 76 (21.35%) | 36 (20.11%) | 40 (22.60%) |  |
| Ground-level fall | 149 (41.85%) | 79 (44.13%) | 70 (39.55%) |  |
| Other | 26 (7.30%) | 13 (7.26%) | 13 (7.34%) |  |
| GCS: |  |  |  | 0.005 |
| Mild | 214 (60.11%) | 121 (67.60%) | 93 (52.54%) |  |
| Moderate | 68 (19.10%) | 32 (17.88%) | 36 (20.34%) |  |
| Severe | 74 (20.79%) | 26 (14.53%) | 48 (27.12%) |  |
| Glu | 7.30 [5.77;9.40] | 6.50 [5.20;8.30] | 7.80 [6.70;9.80] | <0.001 |
| Pupil_reaction: |  |  |  | 0.079 |
| No | 297 (83.43%) | 156 (87.15%) | 141 (79.66%) |  |
| Yes | 59 (16.57%) | 23 (12.85%) | 36 (20.34%) |  |
| Hematoma_type: |  |  |  | <0.001 |
| tICH | 170 (47.75%) | 60 (33.52%) | 110 (62.15%) |  |
| tSDH | 64 (17.98%) | 32 (17.88%) | 32 (18.08%) |  |
| tEDH | 54 (15.17%) | 31 (17.32%) | 23 (12.99%) |  |
| tSAH | 68 (19.10%) | 56 (31.28%) | 12 (6.78%) |  |
| multiple_hematomas: |  |  |  | <0.001 |
| No | 178 (50.00%) | 133 (74.30%) | 45 (25.42%) |  |
| Yes | 178 (50.00%) | 46 (25.70%) | 132 (74.58%) |  |
| Hematoma_heterogeneity: |  |  |  | <0.001 |
| No | 271 (76.12%) | 152 (84.92%) | 119 (67.23%) |  |
| Yes | 85 (23.88%) | 27 (15.08%) | 58 (32.77%) |  |
| White_blood_cell_count | 13.31 [9.87;16.67] | 11.85 [9.22;15.57] | 15.17 [11.15;18.06] | <0.001 |
| Monocyte_count | 0.69 [0.51;0.94] | 0.59 [0.45;0.84] | 0.79 [0.60;1.06] | <0.001 |
| Lymphocyte_count | 0.95 [0.69;1.29] | 1.00 [0.79;1.39] | 0.88 [0.60;1.18] | 0.004 |
| MLR | 0.74 [0.48;1.10] | 0.62 [0.39;0.90] | 0.94 [0.62;1.35] | <0.001 |
| Plt | 176.50 [145.00;210.25] | 181.00 [150.00;218.00] | 166.00 [140.00;201.00] | 0.003 |
| Ca | 2.17 [2.07;2.28] | 2.21 [2.14;2.31] | 2.10 [2.01;2.24] | <0.001 |
| Ka | 3.67 [3.38;3.92] | 3.71 [3.40;3.93] | 3.62 [3.25;3.90] | 0.120 |
| PT | 11.40 [10.70;12.30] | 11.30 [10.60;12.30] | 11.50 [10.80;12.40] | 0.403 |
| APTT | 25.40 [23.10;28.30] | 25.50 [23.10;28.90] | 25.20 [23.10;27.70] | 0.332 |
| INR | 0.96 [0.89;1.02] | 0.96 [0.89;1.01] | 0.97 [0.90;1.03] | 0.161 |
| FIB | 2.32 [1.94;3.01] | 2.33 [1.98;3.02] | 2.30 [1.91;3.00] | 0.266 |
| DD | 9.95 [3.27;22.57] | 5.80 [1.65;12.85] | 15.50 [7.50;33.30] | <0.001 |

**Table S2. Variables selected by LASSO regression with corresponding coefficients at Lambda.1se**

| **Variable** | **Coefficient (β)** |
| --- | --- |
| Intercept | 2.4040 |
| Age | 0.0067 |
| Smoking history | 0.3461 |
| Hematoma type | -0.1746 |
| Multiple hematomas | 0.8946 |
| MLR | 0.4044 |
| Calcium | -1.6408 |
| D-dimer | 0.0151 |

**Table S3. Variance inflation factor (VIF) values for variables included in the multivariate logistic regression model.**

|  | **VIF** |
| --- | --- |
| age | 1.020341744099451 |
| smoke | 1.0365898702546 |
| Hematoma_type | 1.132588212609568 |
| multiple_hematomas | 1.189618131764874 |
| MLR | 1.047758034147258 |
| Ca | 1.021750102247885 |
| DD | 1.092148196950475 |

**Table S4.** Comparison of AUC values of five machine learning models in the training cohort.

|  | DT | LM | XGBOOST | svm | lgbm |
| --- | --- | --- | --- | --- | --- |
| DT | 1 | 0.349482 | 0.017121 | 0.091248 | 2.9E-06 |
| LM | 0.349482 | 1 | 0.016924 | 0.953461 | 0.000156 |
| XGBOOST | 0.017121 | 0.016924 | 1 | 9.00E-07 | 2.15E-09 |
| svm | 0.091248 | 0.953461 | 9.00E-07 | 1 | 0.000193 |
| lgbm | 2.9E-06 | 0.000156 | 2.15E-09 | 0.000193 | 1 |

**Table S5.** Comparison of AUC values of five machine learning models in the validation cohort.

|  | DT | LM | XGBOOST | svm | lgbm |
| --- | --- | --- | --- | --- | --- |
| DT | 1 | 0.154945 | 0.619466 | 0.02156 | 0.182913 |
| LM | 0.154945 | 1 | 0.290421 | 0.893206 | 0.006256 |
| XGBOOST | 0.619466 | 0.290421 | 1 | 0.041721 | 0.087472 |
| svm | 0.02156 | 0.893206 | 0.041721 | 1 | 0.004383 |
| lgbm | 0.182913 | 0.006256 | 0.087472 | 0.004383 | 1 |

**Table S6.** Precision–recall (PR) parameters of five machine learning models in the training cohort.

| dataset | PRAUC | type |
| --- | --- | --- |
| dev | 0.447 | DT |
| dev | 0.451 | lgbm |
| dev | 0.451 | LR |
| dev | 0.451 | SVM |
| dev | 0.451 | xgboost |

**Table S7.** Precision–recall (PR) parameters of five machine learning models in the validation cohort.

| dataset | PRAUC | type |
| --- | --- | --- |
| vad | 0.449 | DT |
| vad | 0.455 | lgbm |
| vad | 0.455 | LR |
| vad | 0.455 | SVM |
| vad | 0.455 | xgboost |

**Table S8.** Brier scores of five machine learning models in the training cohort.

| name | bs_score |  |  |
| --- | --- | --- | --- |
| xgboost | brier_score= 0.1050 (0.0872-0.1267) | | |
| SVM | brier_score= 0.1481 (0.1275-0.1723) | | |
| LR | brier_score= 0.1426 (0.1193-0.1710) | | |
| lgbm | brier_score= 0.2441 (0.2426-0.2458) | | |
| DT | brier_score= 0.1199 (0.0945-0.1483) | | |

**Table S9.** Brier scores of five machine learning models in the validation cohort.

| name | bs_score |  |  |  |
| --- | --- | --- | --- | --- |
| xgboost | brier_score= 0.1400 (0.1089-0.1926) | | | |
| SVM | brier_score= 0.1261 (0.1027-0.1528) | | | |
| LR | brier_score= 0.1174 (0.0896-0.1530) | | | |
| lgbm | brier_score= 0.2426 (0.2404-0.2451) | | | |
| DT | brier_score= 0.1544 (0.1107-0.2142) | | | |

Table S10. Area under the ROC curve (AUC) with 95% confidence intervals for five machine learning models in the training and validation cohorts.

| model | dfclass | AUC | Low | Up |
| --- | --- | --- | --- | --- |
| LR | dev | 0.873 | 0.829 | 0.917 |
| DT | dev | 0.901 | 0.862 | 0.939 |
| XGB | dev | 0.937 | 0.908 | 0.966 |
| SVM | dev | 0.871 | 0.827 | 0.915 |
| LGBM | dev | 0.728 | 0.667 | 0.788 |
| LR | vad | 0.921 | 0.872 | 0.969 |
| DT | vad | 0.858 | 0.787 | 0.929 |
| XGB | vad | 0.876 | 0.811 | 0.942 |
| SVM | vad | 0.925 | 0.878 | 0.972 |
| LGBM | vad | 0.783 | 0.698 | 0.867 |

**Table S 11. Diagnostic performance of the nomogram for predicting early intracranial hematoma progression.**

| datatset | Cutoff | AUC | AUC.SE | AUC.low | AUC.up | P |
| --- | --- | --- | --- | --- | --- | --- |
| Trianset_raw | 0.353 | 0.873 | 0.022 | 0.829 | 0.917 | ＜0.001 |
| Trianset_binary | 0.353 | 0.812 | 0.033 | 0.747 | 0.877 | ＜0.001 |
| Validationset_raw | 0.413 | 0.921 | 0.025 | 0.872 | 0.969 | ＜0.001 |
| Validationset_binary | 0.353 | 0.814 | 0.048 | 0.72 | 0.907 | ＜0.001 |

**Table S12. Diagnostic performance of the nomoscore in predicting early intracranial hematoma progression. Metrics include AUC, sensitivity, specificity, PPV, and NPV in training and validation cohorts.**

| datatset | Cutoff | AUC | AUC.SE | AUC. low | AUC. up | P |
| --- | --- | --- | --- | --- | --- | --- |
| Trainset | 138.369 | 0.873 | 0.022 | 0.829 | 0.917 | ＜0.001 |
| Valiset | 138.369 | 0.814 | 0.048 | 0.72 | 0.907 | ＜0.001 |
